# Maternal immune activation in rats induces dysfunction of placental leucine transport and alters fetal brain growth

**DOI:** 10.1042/CS20220245

**Published:** 2022-08-05

**Authors:** Hager M. Kowash, Harry G. Potter, Rebecca M. Woods, Nick Ashton, Reinmar Hager, Joanna C. Neill, Jocelyn D. Glazier

**Affiliations:** 1 Division of Developmental Biology and Medicine, School of Medical Sciences, Faculty of Biology, Medicine and Health, Manchester Academic Health Science Centre, University of Manchester, Manchester M13 9WL, U.K.; 2 Division of Evolution, Infection and Genomics, School of Biological Sciences, Faculty of Biology, Medicine and Health, Manchester Academic Health Science Centre, University of Manchester, Manchester M13 9PT, U.K.; 3 Division of Cardiovascular Sciences, School of Medical Sciences, Faculty of Biology, Medicine and Health, Manchester Academic Health Science Centre, University of Manchester, Manchester M13 9PT, U.K.; 4 Division of Pharmacy and Optometry, School of Health Sciences, Faculty of Medicine, Biology and Health, Manchester Academic Health Science Centre, University of Manchester, Manchester M13 9PT, U.K.

**Keywords:** Amino acid, cytokines, placenta, poly(I:C), pregnancy, system L

## Abstract

Maternal infection during pregnancy increases the offspring risk of developing a variety of neurodevelopmental disorders (NDDs), including schizophrenia. While the mechanisms remain unclear, dysregulation of placental function is implicated.

We hypothesised that maternal infection, leading to maternal immune activation and stimulated cytokine production, alters placental and yolk sac amino acid transport, affecting fetal brain development and thus NDD risk. Using a rat model of maternal immune activation induced by the viral mimetic polyinosinic:polycytidylic acid (poly(I:C)), we investigated placental and yolk sac expression of system L amino acid transporter subtypes which transport several essential amino acids including branched-chain amino acids (BCAA), maternal and fetal BCAA concentration, placental ^14^C-leucine transport activity and associated impacts on fetal growth and development.

Poly(I:C) treatment increased acutely maternal IL-6 and TNFα concentration, contrasting with IL-1β. Transcriptional responses for these pro-inflammatory cytokines were found in placenta and yolk sac following poly(I:C) treatment. Placental and yolk sac weights were reduced by poly(I:C) treatment, yet fetal body weight was unaffected, while fetal brain weight was increased. Maternal plasma BCAA concentration was reduced 24 h post-poly(I:C) treatment, yet placental, but not yolk sac, BCAA concentration was increased. Placental and yolk sac gene expression of *Slc7a5, Slc7a8* and *Slc43a2* encoding LAT1, LAT2 and LAT4 transporter subtypes, respectively, was altered by poly(I:C) treatment. Placental ^14^C-leucine transport was significantly reduced 24 h post-treatment, contrasting with a significant increase 6 days following poly(I:C) treatment.

Maternal immune activation induces dysregulated placental transport of amino acids affecting fetal brain development, and NDD risk potential in offspring.

## Introduction

Epidemiological studies have shown that maternal exposure to infection during pregnancy contributes to the aetiology of neurodevelopmental diseases (NDDs) in the offspring, including schizophrenia (SZ) [1–3], estimated to affect ∼0.4–1.0% of the global population [4]. Different types of microbial infections during pregnancy are known to cause impaired neurodevelopment, neuroanatomical abnormalities, altered behaviour and cognitive deficits reminiscent of NDDs [1–3]. This suggests that it is the response to infection, rather than the infectious pathogen *per se*, that links exposure to maternal infection *in utero* to increased risk of NDDs [1–3,5–9]. Understanding the underlying mechanisms is a major focus of research efforts, but significant gaps remain, in particular how maternofetal nutrient transport function is impacted. To investigate this, we focus here on the placental and yolk sac transport of the essential amino acid leucine in a rat model of maternal immune activation (mIA) simulating viral infection during pregnancy.

Studies of seasonal influenza epidemics have shown that the risk of developing SZ increases 7-fold if exposure occurred in the first trimester, with a 3-fold SZ risk reported following influenza exposure during mid-pregnancy [10]. However, if infection occurred later on in the second and third trimesters, risk was not appreciably greater than in unaffected individuals [10]. This suggests that the period of early-mid pregnancy is a particularly important developmental window during which the susceptibility of neurogenesis and neurodevelopmental processes to perturbation is associated with increased SZ risk [5,11,12]. Hence, the neurodevelopmental hypothesis of SZ proposes that the propensity to develop SZ arises from the disrupted programmed maturation of the brain in prenatal and early neonatal life, which leads to enduring changes in behavioural development and increased risk of developing SZ during late adolescence or early adulthood [11]. Specifically, mIA-evoked cytokine signalling has been shown to elicit effects on neurodevelopmental processes with ensuing impacts on brain function [6,13,14]. Such a hypothesis is also consistent with altered expression of genes related to immune function and inflammatory markers in SZ-affected individuals [9,14]. The mechanisms through which maternal infection leads to altered offspring brain development and increased risk of developing NDDs have, however, remained elusive and are now the focus of significant research effort using animal models.

Several animal models, mainly in rodents [6,15,16] but also non-human primates [17], have been developed to simulate the inflammatory cytokine paradigm of NDDs. A variety of immunostimulants such as influenza virus, bacterial endotoxin lipopolysaccharide (LPS) and the synthetic dsRNA viral mimetic polyinosinic:polycytidylic acid (poly(I:C)) have been used to model different modes of infection. Rodent models of mIA, including both mouse [7,16,18] and rat [7,16,18–24], demonstrate neuroanatomical, neurochemical, transcriptomic and epigenomic alterations in offspring brain regions, together with a range of aberrant behavioural traits reminiscent of SZ.

Our previous work has shown that poly(I:C) administration at gestational day (GD) 15 resulted in a significantly reduced placental weight at GD21 whilst fetal weight was unaffected [25]. This led us to hypothesise that placental nutrient transport efficiency must be altered to maintain fetal weight in the face of a reduced placental weight. In the present study, we focus on the system L amino acid transporter. System L transports essential amino acids, including branched-chain amino acids (BCAA), and its Na^+^-independent exchanger activity relies on the accumulative uptake of neutral amino acids mediated by system A transporter activity, which it then uses as exchange substrates [26,27]. This coupling of system A and system L amino acid transporter activities in providing neutral amino acids, and importantly essential amino acids, is crucial to support fetal protein synthesis, metabolism, development and growth. Impairment of either transport mechanism is associated with fetal growth restriction [26,28]. LAT1 and LAT2 constitute the light chains of system L which each form a heterodimeric complex with the heavy chain CD98 to mediate system L activity, although it is the LAT1 and LAT2 light chains that confer catalytic activity [26,27].

Whilst nutrient transport across the rat placenta supports the dynamic rates of fetal growth and development over the mid-late period of rat pregnancy, the rat visceral yolk sac (hereafter referred to as yolk sac) is likely to play a key role in fetal nutrient provision during the early phases of pregnancy before the full establishment of placental nutrient transport function, although it also functions to augment fetal growth in late gestation [29]. Such a yolk sac contribution appears to be particularly important in providing amino acids to the developing fetus [30,31]. Consistent with these concepts, we have recently shown that the rat yolk sac expresses LAT1 and LAT2 subtypes of the system L amino acid transporter together with the system L-type transporter LAT4, in common with the rat placenta [32]. Although LAT4 (*Slc43a2*) is a monomeric, facilitated transporter with a mode of transport distinct to that of system L-mediated amino acid exchange activity, it can be regarded as a system L-type transporter because it transports large, essential neutral amino acids in a Na^+^-independent manner with overlapping amino acid substrate selectivity to LAT1 and LAT2 subtypes of system L, and is also inhibited by the classic system L inhibitor 2-aminobicyclo [2.2.1] heptane-2-carboxylic acid [BCH; 27, 32]. The distribution of these three LAT proteins to the rat yolk sac [32] implicates a role for the yolk sac in the transport of essential amino acids to the developing embryo when neurogenesis is occurring. However, the combined effects of poly(I:C)-induced mIA on transporter expression and activity in both the placenta and yolk sac remain unknown.

To address this significant gap in our mechanistic understanding of how maternal infection influences fetal development and later function, here we investigate predicted mechanistic links between mIA, altered placental amino acid transport function and fetal growth and developmental outcomes. First, our focus on system L amino acid transporter expression and function is based on its critical role as a transport mechanism of essential amino acids required for fetal growth and development [26,28], as well as its major role in providing amino acid precursors for neurotransmission and neuromodulation in the brain [33,34]. Second, we hypothesised that any adaptive responses in altered brain development may arise through altered fetal amino acid provision, and have therefore investigated both acute (24 h) and longer-term (6 days; 144 h) effects following induction of mIA by poly(I:C) on: (i) fetal developmental and growth outcomes, (ii) system L subtype expression in fetus-matched placenta and yolk sac, (iii) tissue BCAA accumulation, and (iv) the maternofetal flux of leucine as a model substrate of system L activity and transported by LAT1, LAT2 and LAT4 proteins. Third, we stratify our results by fetal sex because several placental responses to mIA induced by poly(I:C) exhibit sex-specificity, which may contribute to the sex-dependency of neurodevelopmental disease risk [35,36]. Hence, the overarching objective of this study was to advance our understanding of the mechanisms that contribute to the developmental programming of NDDs and the etiopathogenesis of disorders such as SZ. This is essential if we are to develop prevention strategies in at risk populations alongside improved therapeutics.

## Materials and methods

### Animals

Virgin female Wistar rats (Charles River, U.K.) weighing between 220 and 315 g (mean 262.8 ± 2.8 g, *N*=63) were acclimatised to the housing conditions for at least one week before mating, maintained at a temperature of 21–23°C, a humidity of 55–60% with a 12 h:12 h light:dark cycle (lights on at 07:00). Female rats were mated with adult male Wistar rats (Charles River, U.K.) and pair-housed in individually ventilated cages with split-level environmental enrichment (GR1800 Double-Decker Cage, Tecniplast, U.K.), with *ad libitum* access to standard rat chow (Special Diet Services, U.K.) and water. Gestational day 1 (GD1) was determined by the presence of a vaginal plug.

### Poly(I:C) treatment

On GD15 (term is GD23), pregnant dams were block randomised using an online random number generator to treatment or vehicle groups. Dams in the treatment group received a single intraperitoneal injection of the viral mimetic poly(I:C) (InvivoGen, San Diego, U.S.A., low molecular weight (LMW) form; catalogue number tlrl-picw, lot numbers PIW-39-01 and PIW-40-01) at a dose of 10 mg/kg body weight [20,25]; control dams were injected with an equivalent volume of vehicle (endotoxin-free 0.9% saline, InvivoGen). Subsequent analyses were not performed in a blinded manner with respect to treatment group. The timeframe over which mIA was induced by poly(I:C) in the present study corresponded to the timing of peak rat cortical neurogenesis at GD14-15, which then declines at GD17 [18,37,38].

### Poly(I:C) source

We used the LMW form of poly(I:C) supplied by InvivoGen to mimic the acute phase of viral infection and evoke the mIA-induced synthesis of pro-inflammatory cytokines. We [25], and others [39], have previously shown that this source of poly(I:C) comprises a much more uniform product consisting of poly(I:C) species of a relatively narrow molecular size distribution with negligible endotoxin contamination, as compared with that obtained from Sigma-Aldrich which has significant endotoxin contamination in comparison [25]. This has the advantage of more consistent immunogenic responses through the specific activation of TLR3 to stimulate pro-inflammatory cytokine production without the potential for contaminating endotoxin to activate TLR4 pathways leading to the exacerbated activation of NF-κB [40], or for varying constituent higher molecular weight poly(I:C) species to potentiate cytokine synthesis leading to more variable maternal immunogenic responses [25,39]. This methodological point remains of concern for mechanistic interpretation as well as mIA model reproducibility, as it may lead to differing neurobehavioural phenotypes in the offspring related to maternal cytokine responses [19,25,39,41–42].

### Measurement of maternal pro-inflammatory cytokines

Maternal blood was collected at 2, 3 and 24 h post-injection from the tail vein and plasma harvested [20]. Maternal plasma IL-6, TNFα and IL-1β concentration was determined using rat-specific ELISAs according to the manufacturer’s instructions (Abcam, Cambridge, U.K.; ab100772, ab100784 and ab100767, respectively).

### Fetal tissue harvesting

Fetal tissues were harvested following treatment at GD15 (3 h), GD16 (24 h) and GD21 (6 days; 144 h) to examine acute and long-term effects. Dams were anaesthetised with 4% isoflurane (Abbott, Maidenhead, U.K.) in oxygen at 2 L/min. Dams were killed by exsanguination, with collection of maternal blood by cardiac puncture under anaesthesia, followed by removal of the heart. After laparotomy, each fetus, placenta and yolk sac were carefully dissected and weighed tissues were stored in RNA later (Sigma-Aldrich, Gillingham, U.K.) or flash-frozen. Litter size and number of resorptions were recorded. Whole fetal brain and liver were dissected and weighed. At GD21, fetal blood was collected from an axillary incision and plasma harvested. Fetuses were killed by decapitation and fetal tail tips were taken for sextyping.

### Fetal sextyping

Genomic DNA from fetal rat tail tips was extracted using the KAPA Express Extract Kit (KAPA Biosystems, Merck, Gillingham, U.K.), according to the manufacturer’s instructions. Male fetuses were positively identified by PCR amplification of the male-specific *Sry* gene with visualisation of *Sry* amplicons on an agarose gel; female sex was identified by the lack of a *Sry* amplicon. Further details, including primer sequences, have been provided previously [32].

### Quantification of placental area

Following harvest, placental tissue samples (GD15, 16 and 21) were fixed in 3 mL 10% neutral buffered formalin and then embedded in paraffin wax. Placental sections (5 μm; Leica RM2245 Semi-automated Rotary Microtome; Leica Biosystems, U.K.) were stained with haematoxylin and eosin (Sigma-Aldrich, U.K.). Whole sections (3 per slide) were imaged using brightfield microscopy (3D-Histech Pannoramic-250 microscope slide-scanner) at the Bioimaging Facility, University of Manchester. Quantification of placental area, comprising the junctional and labyrinth zones, was performed using ImageJ (NIH, U.S.A.) at 1× magnification per section.

### BCAA concentration in plasma and tissue lysates

At GD16 and GD21, plasma and tissue BCAA concentration was measured using a BCAA assay kit (MAK003; Sigma-Aldrich, U.K.), according to the manufacturer’s instructions.

### RNA extraction, cDNA synthesis and quantitative PCR for target genes

Total RNA was extracted from placental and yolk sac tissues using RNeasy Plus kit (Qiagen, Manchester, U.K.) according to the manufacturer’s instructions, and 2 μg RNA used to generate cDNA (Quantitect Reverse Transcription Kit; Qiagen, U.K.). Expression of several gene candidates was quantified in duplicate using validated primers (QuantiTect Primer Assays, Qiagen, U.K.; Supplementary Table S1) with the Quantifast SYBR Green PCR Kit (Qiagen, U.K.) using a Stragene Mx3000P qPCR machine. Reference gene stability was determined using primers encoding several candidate reference genes: *Ubc, Actb, Ywhaz, Gapdh, Mdh1* and *B2m* (rat SYBRgreen reference gene detection kit, *HK-SY-ra*; Primerdesign, Chandler’s Ford, U.K.) followed by geNorm analysis (qBase+ software; Biogazelle, Belgium). Expression of *Gapdh* was the most stable across all tissues, conditions and sexes, and was thus used for gene expression normalisation.

### System L amino acid transporter subtype expression

Placenta and yolk sac tissue lysates were prepared from frozen tissue collected on GD16, and the expression of LAT1, LAT2 and LAT4 system L amino acid transporter subtypes (normalised to the reference protein β-actin) determined. Further details of the Western blotting protocol and antibodies used have been reported previously [32]. Preliminary experiments indicated that LAT1 expression was undetectable or barely detectable in yolk sac at comparable protein loadings to placental lysates, in agreement with our previous findings [32], and therefore LAT1 expression in yolk sac was excluded from further analysis.

### Maternofetal transport of ^14^C-leucine

Maternofetal transport of ^14^C-leucine, as an index of placental system L amino acid transporter activity, was determined using ^14^C-leucine as a model amino acid substrate of system L activity [27]. Unidirectional maternofetal transport of ^14^C-leucine across the intact placenta *in vivo* was measured on GD16 and GD21, 24 h and 6 days following poly(I:C) treatment, respectively. The pregnant dam was anaesthetised with 4% isoflurane in oxygen at 2 L/min followed by an injection of 100 mg/kg body weight Inactin (Sigma-Aldrich, U.K.). The maternal carotid artery and jugular vein were cannulated. ^14^C-leucine (0.185 MBq/200 µl; NEC279E250UC Perkin Elmer, Beaconsfield, U.K.) in 0.9% saline was injected into the jugular vein at time zero, followed by sequential collection of maternal blood from the carotid artery every 15 s for 2 min (GD16) or every 8 s for 48 s (GD21), after which the experiment was terminated, and the dam was killed by cervical dislocation. This time course was selected to ensure that the fetomaternal backflux of ^14^C-leucine was minimal. Fetuses, placentas and yolk sacs were harvested, weighed and ^14^C-leucine content of fetal tissues was counted, after removal of fetal tail tip for sextyping. Maternofetal transport of ^14^C-leucine, an index of placental system L transport capacity, was calculated as the fetal accumulation of ^14^C-leucine normalised to placental weight, as described previously [43].

### Statistics

Statistical analysis was conducted using SPSS (version 25). General Linear Models (GLM) and General Linear Mixed Models (GLMM) were used to analyse data, with treatment group and sex as fixed factors for outcome variables, and dam as a random factor where appropriate. Sex-specific effects were investigated by analysing the interaction between treatment and sex. Placental, yolk sac and fetal body and organ weights, gene expression, BCAA concentration and ^14^C-leucine accumulation were analysed individually nested within the dam as a random factor. Correlations between fetal and tissue weights were analysed by Pearson correlation analysis. Data are presented as mean + SEM (GraphPad Prism 7 software, U.S.A.). For all analyses, *N* refers to the number of litters (or dams) and *n* is the number of individual fetuses or individual fetal tissues.

## Results

### Poly(I:C) induces maternal immune activation and pro-inflammatory cytokine responses

**Figure 1:**
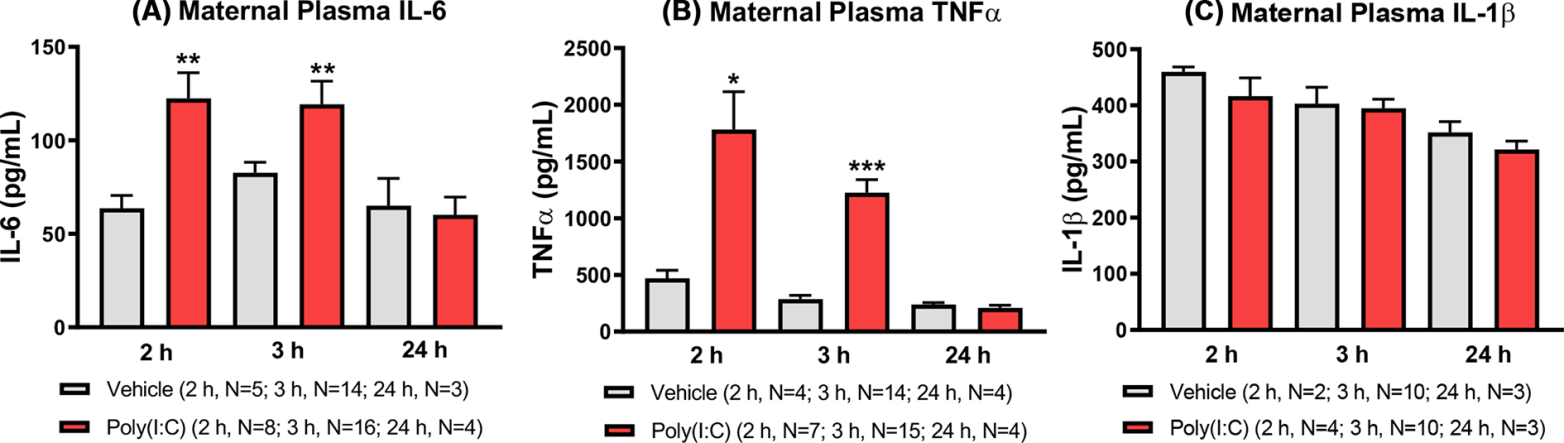
Maternal plasma concentration of pro-inflammatory cytokines (A) IL-6, (B) TNFα and (C) IL-1β at 2, 3 and 24 h post-poly(I:C) treatment The acute rise in the maternal plasma concentration of pro-inflammatory cytokines IL-6 and TNF-α confirms the induction of mIA following poly(I:C) treatment. Data are presented as mean + SEM; *P<0.05, **P<0.01, ***P<0.001 vs vehicle control at same time point.

Poly(I:C) was effective in eliciting acute maternal pro-inflammatory cytokine responses, with the elevations in IL-6 and TNFα plasma concentrations occurring over a similar timeframe following poly(I:C) treatment (Figure 1A,B). At 2 h, IL-6 concentration was significantly elevated in poly(I:C)-treated dams (GLM; *F*_(1,11)_ = 10.10, *P*=0.009; Figure 1A) compared with vehicle-treated dams and remained significantly higher at 3 h post-treatment (GLM; *F*_(1,29)_ = 7.88, *P*=0.009; Figure 1A). Similarly, TNFα was also significantly increased in poly(I:C) dams at 2 h (GLM; *F*_(1,9)_ = 8.31, *P*=0.018; Figure 1B) and 3 h (GLM; *F*_(1,27)_ = 47.06, *P*<0.001; Figure 1B). The magnitude of response (mean fold-change relative to vehicle controls) was greater for TNFα, being 3.8- and 4.3-fold at 2 and 3 h, respectively, as compared with 1.9- and 1.4-fold for IL-6. However, by 24 h post-treatment, the concentration of both IL-6 and TNFα had declined to control levels (Figure 1A,B). In contrast, maternal IL-1β concentration remained relatively stable over the same timeframe, with concentrations unchanged compared with vehicle control at each of the measured time points (Figure 1C).

### Poly(I:C) reduces litter size in late gestation but has no effect earlier in gestation

Poly(I:C) treatment (administered at GD15) was tolerated well by the pregnant rat dams with subsequent maintenance of pregnancy (Table 1). Poly(I:C) treatment did result in a significantly increased number of resorptions at 3 h after treatment (GLM; F_(1,12)_ = 6.86, *P*=0.022), but this was not evident 24 h after treatment and was insufficient to impact on litter size at these gestational ages (Table 1). However, 6 days after mIA induction, we found only a non-significant trend towards an increased number of resorptions (GLM; *F*_(1,20)_ = 3.36, *P*=0.082), which may contribute to the significant reduction in litter size observed in the poly(I:C) group at this stage of gestation (GLM; *F*_(1,20)_ = 4.95, *P*=0.038). Poly(I:C) treatment had no effect on fetal sex distribution between treatment groups at any gestational age (Table 1).

**Table 1:** Fetal characteristics, fetal organ, placental and yolk sac weights on GD15, GD16 and GD21

Gestational day (hours post-treatment)	GD15 (3 h)	GD16 (24 h)	GD21 (144 h)
**No. of resorptions**			
Vehicle	0.5 ± 0.2 [*N*=6]	1.4 ± 0.4 [*N*=13]	1.3 ± 0.3 [*N*=9]
Poly(I:C)	2.0 ± 0.5* [*N*=8]	1.9 ± 0.3 [*N*=13]	3.6 ± 1.0 [*N*=13]
**Litter size**			
Vehicle	15.2 ± 1.2 [*N*=6]	13.4 ± 0.8 [*N*=13]	15.3 ± 0.9 [*N*=9]
Poly(I:C)	14.6 ± 0.9 [*N*=8]	15.2 ± 0.4 [*N*=13]	12.5 ± 0.9* [*N*=13]
**% Male**			
Vehicle	56.3 ± 6.8 [*N*=6]	49.6 ± 3.7 [*N*=13]	49.4 ± 3.7 [*N*=9]
Poly(I:C)	50.9 ± 3.9 [*N*=8]	50.9 ± 4.1 [*N*=13]	53.1 ± 4.3 [*N*=13]
**% Female**			
Vehicle	43.7 ± 6.8 [*N*=6]	50.5 ± 3.7 [*N*=13]	50.6 ± 3.7 [*N*=9]
Poly(I:C)	49.2 ± 3.9 [*N*=8]	49.1 ± 4.1 [*N*=13]	47.0 ± 4.3 [*N*=13]
**Fetal weight (g)**			
Vehicle	0.1568 ± 0.0015 (*n*=86)	0.2538 ± 0.0014 (*n*=170)	3.6410 ± 0.0411 (*n*=127)
Poly(I:C)	0.1535 ± 0.0015 (*n*=113)	0.2535 ± 0.0016 (*n*=187)	3.5720 ± 0.0201 (*n*=155)
**Placenta weight (g)**			
Vehicle	0.1589 ± 0.0033 (*n*=86)	0.2432 ± 0.0036 (*n*=165)	0.5015 ± 0.0059 (*n*=132)
Poly(I:C)	0.1680 ± 0.0038 (*n*=109)	0.2238 ± 0.0035‡ (*n*=190)	0.4862 ± 0.0067 (*n*=152)
**Yolk sac weight (g)**			
Vehicle	0.0179 ± 0.0005 (*n*=86)	0.0301 ± 0.0005 (*n*=169)	0.1091 ± 0.0017 (*n*=130)
Poly(I:C)	0.0166 ± 0.0004* (*n*=112)	0.0292 ± 0.0004 (*n*=187)	0.1019 ± 0.0020† (*n*=153)
**Brain weight (g)**			
Vehicle	0.0576 ± 0.0009 (*n*=84)	0.0903 ± 0.0011 (*n*=92)	0.1605 ± 0.0013 (*n*=69)
Poly(I:C)	0.0598 ± 0.0008* (*n*=113)	0.0950 ± 0.0009† (*n*=75)	0.1643 ± 0.0010* (*n*=74)
**Liver weight (g)**			
Vehicle	0.0041 ± 0.0002 (*n*=84)	0.0114 ± 0.0002 (*n*=89)	0.2713 ± 0.0054 (*n*=70)
Poly(I:C)	0.0042 ± 0.0001 (*n*=110)	0.0124 ± 0.0003 (*n*=91)	0.2610 ± 0.0051 (*n*=74)
**Fetal:placental weight ratio**			
Vehicle	0.9932 ± 0.0231 (*n*=89)	1.0640 ± 0.0161 (*n*=168)	7.0690 ± 0.0955 (*n*=133)
Poly(I:C)	0.9357 ± 0.0205 (*n*=110)	1.1370 ± 0.0182† (*n*=191)	7.3520 ± 0.0955* (*n*=158)
**Brain:liver weight ratio**			
Vehicle	15.4800 ± 0.6706 (*n*=84)	8.1290 ± 0.1900 (*n*=92)	0.5975 ± 0.0097 (*n*=69)
Poly(I:C)	15.2300 ± 0.5796 (*n*=110)	8.2210 ± 0.1867 (*n*=77)	0.6314 ± 0.0129* (*n*=76)
**Brain:body weight ratio**			
Vehicle	0.3740 ± 0.0043 (*n*=84)	0.3622 ± 0.0034 (*n*=94)	0.0443 ± 0.0004 (*n*=69)
Poly(I:C)	0.4063 ± 0.0064‡ (*n*=114)	0.3877 ± 0.0033‡ (*n*=78)	0.0472 ± 0.0004‡ (*n*=74)

Data are presented as mean ± SEM; *N* = no. of dams, *n* = no. of fetuses; *P<0.05, †*P*<0.01, ‡*P*<0.001 vs. vehicle control.

### Poly(I:C) has no effect on fetal body weight, but fetal brain weight is significantly increased

Our analysis of individual fetal body weights using a GLM model with a nested-within-dam design (GLMM) at GD15, 16 and 21, demonstrated that poly(I:C) administration had no significant effect on fetal body weight compared with vehicle at each of the gestational time points (Table 1). Fetal weight in both sexes increased significantly as gestation advanced (Table 1) regardless of treatment group (GLMM; *F*_(2,833)_ = 24641.75, *P*<0.001), and both groups exhibited a similar fetal weight gain between GD15 and GD21, with a 23.3-fold increase in the poly(I:C) group, as compared with 23.2-fold increase the vehicle group (Table 1).

Male fetuses were significantly heavier than females across both treatment groups at each gestational stage, as expected [44]; GD15 (GLMM; *F*_(1,195)_ = 5.46, *P*=0.020, Supplementary Figure S1A), GD16 (GLMM; *F*_(1,353) =_ 29.59, *P*<0.001, Supplementary Figure S1B) and GD21 (GLMM; *F*_(1,278)_ = 19.93, *P*<0.001, Supplementary Figure S1C).

Although fetal body weight was unaffected by poly(I:C) treatment, fetal brain weights were significantly heavier in the poly(I:C) group compared with vehicle at GD15 (GLMM; *F*_(1,196)_ = 4.08, *P*=0.045), GD16 (GLMM; *F*_(1,163)_ = 9.36, *P*=0.003) and GD21 (GLMM; *F*_(1,139)_ = 5.59, *P*=0.019; Table 1). Hence, the ratio of fetal brain weight:body weight was significantly increased in the poly(I:C) group at all gestational time points post-poly(I:C) treatment compared with vehicle-treated controls (Table 1). To determine whether this was associated with enhanced sparing of fetal brain growth at the expense of abdominal organ growth [45], we calculated the fetal brain:liver weight ratio (Table 1). At GD15, fetal brain:liver weight ratio was unaffected by treatment; however, the interaction between treatment and sex showed a trend towards significance (GLMM; *F*_(1,191)_ = 3.35, *P*=0.069), with a marginal trend effect of reduced brain:liver weight ratio in female fetuses of poly(I:C)-treated dams. At GD16, poly(I:C) treatment had no significant effect on the fetal brain:liver weight ratio. Conversely, at GD21, the fetal brain:liver weight ratio was significantly increased in the fetuses of poly(I:C)-treated dams (GLMM; *F*_(1,141)_ = 4.26, *P*=0.041; Table 1).

### Poly(I:C) alters placental and yolk sac weight in a gestation-dependent manner

We measured placental and yolk sac weights as a proxy of their tissue growth. As shown in Table 1, placental and yolk sac tissue weights significantly increased with advancing gestation (placenta, GLMM; *F*_(2,829)_ = 2448.67, *P*<0.001; yolk sac, GLMM; *F*_(2,834)_ = 3056.53, *P*<0.001). Placental weight was unaffected by poly(I:C) treatment at GD15 (3 h post-treatment), whereas at GD16, a day following treatment, placentas from dams exposed to poly(I:C) were significantly lighter compared with vehicle control (GLMM; *F*_(1,351)_ = 15.59, *P*<0.001; Table 1), with a non-significant trend towards being lighter in the poly(I:C) group at GD21 (GLMM; *F*_(1,280)_ = 3.43, *P*=0.065). This was also supported by measurements of total placental area (Supplementary Figure S2), which showed that poly(I:C) administration had no significant effect on total placental area at GD15 (GLMM; *F*_(1,11)_ = 0.19, *P*=0.67) or GD21 (GLMM; *F*_(1,8)_ = 0.37, *P*=0.56). By contrast, total area was significantly reduced in the poly(I:C) treated group at GD16 (GLMM; *F*_(1,6)_ = 9.73, *P*=0.021, Supplementary Figure S2).

The fetal:placental weight ratio, taken as an index of placental transport capacity [46], was significantly increased in the poly(I:C) group at GD16 and GD21 compared to vehicle control (GD16, GLMM; *F*_(1,355)_ = 9.13, *P*=0.003; GD21, GLMM; *F*_(1,287)_ = 4.30, *P*=0.039), whereas there was no significant effect of treatment at GD15 (Table 1).

Whilst there was no significant correlation between fetal and placental weight in either males (*r* = −0.11, *P*=0.47) or females (*r* = 0.14, *P*=0.43) at GD15 (Supplementary Figure S3A), the male group showed a significant relationship (*r* = 0.27, *P*=0.02) at GD16, whereas females did not (*r* = 0.12, *P*=0.26; Supplementary Figure S3B). However, at GD21, there was a highly significant positive correlation between fetal and placental weights in both males (*r* = 0.35, *P*=0.006) and females (*r* = 0.46, *P*<0.001; Supplementary Figure 3C).

The yolk sac exhibited a different pattern of weight changes compared with placenta. Poly(I:C) treatment caused a decrease in yolk sac weight at GD15 (GLMM; *F*_(1,194)_ = 4.13, *P*=0.044), whereas at GD16, there was no significant difference between treatment groups (Table 1). However, at GD21, poly(I:C) treatment induced a reduction in yolk sac weight (GLMM; *F*_(1,280)_ = 8.62, *P*=0.004; Table 1).

Neither fetal sex exhibited a significant relationship between placental and yolk sac weights at GD15 (male, *r* = −0.18,  *P*=0.24; female, *r* = −0.04,  *P*=0.81; Supplementary Figure S3D). Similarly, at GD16 (*r* = 0.17, *P*=0.16; Supplementary Figure S3E) and GD21 (*r* = −0.014,   *P*=0.92; Supplementary Figure S3F), male fetuses showed no significant correlation between the two tissue weights. Conversely, females at GD16 displayed a significant correlation between placenta and yolk sac weight (*r* = 0.48, *P*<0.0001; Figure 3E) and showed a trend towards correlation between placental and yolk sac weight at GD21 (*r* = 0.24, *P*=0.063; Supplementary Figure S3F).

### Poly(I:C) induces changes in toll-like receptor 3 gene expression in both placenta and yolk sac dependent on gestational stage

**Figure 2:**
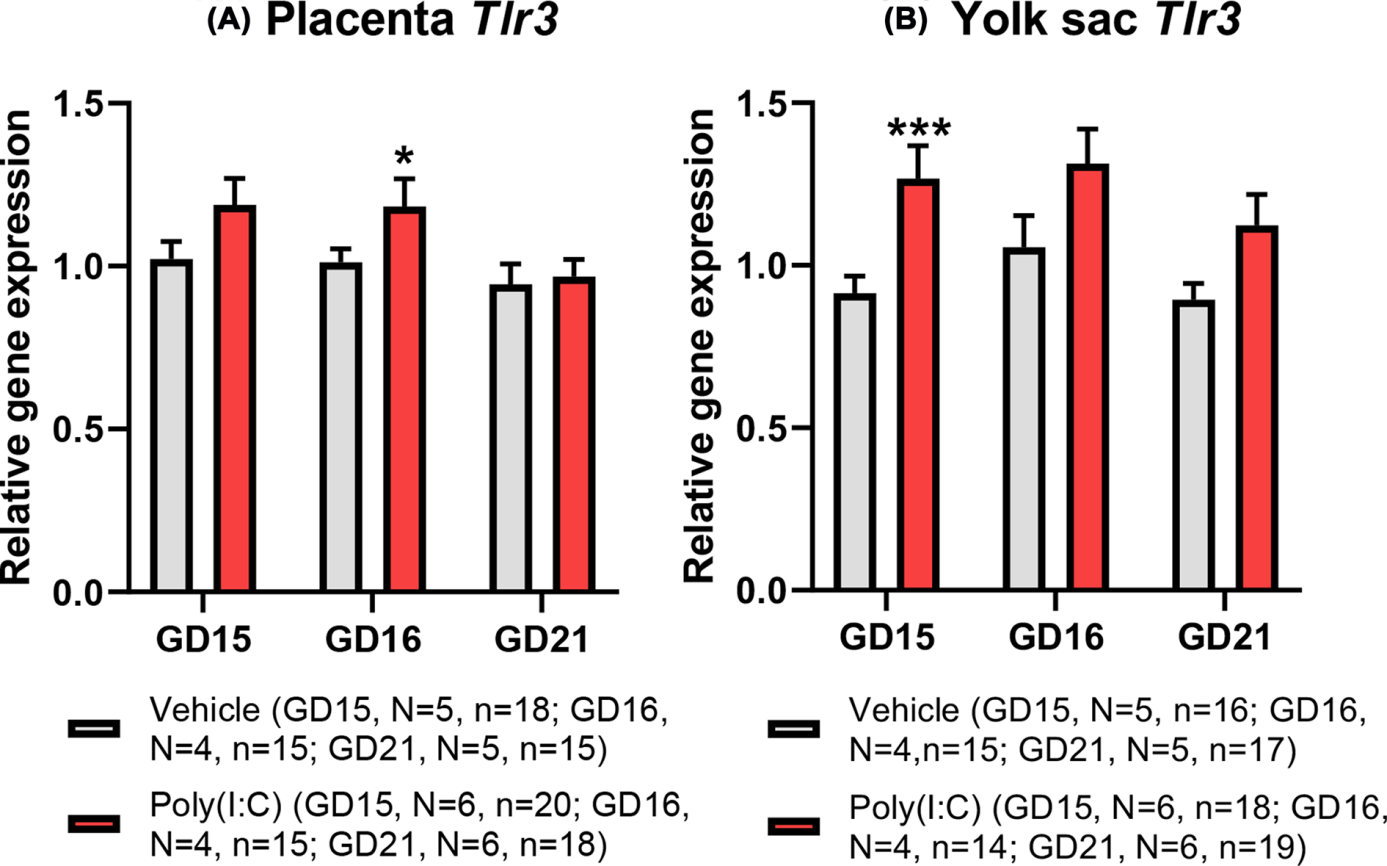
Effect of poly(I:C) treatment on mRNA expression of *Tlr3* at GD15 (3 h post-treatment), GD16 (24 h post-treatment) and GD21 (6 days post-treatment) in (A) placenta and (B) yolk sac Both tissues show stimulated *Tlr3* transcription following poly(I:C) treatment, albeit with a different temporal pattern of response. Data presented as mean + SEM. *P<0.05, ***P<0.001 vs vehicle on same gestational day.

Previous studies in rodents have demonstrated that poly(I:C), used here as a viral dsRNA mimetic, induces placental *Tlr3* gene and TLR3 protein expression [47,48]. TLR3 is the endosomal receptor activated by viral dsRNA ligands to mediate transcriptional responses to viral infection [49]. However, whether the yolk sac expresses *Tlr3* and responds in a similar manner is unknown. We demonstrate that both the placenta and yolk express *Tlr3* mRNA in fetus-matched tissues at all gestational ages examined (Figure 2A,B). Furthermore, *Tlr3* gene expression within each tissue was not altered by advancing gestation. Whilst the placenta demonstrated no treatment effect on *Tlr3* expression at 3 h post-treatment (GD15, Figure 2A), expression of *Tlr3* in the yolk sac was significantly increased in the poly(I:C) group compared with vehicle control (GLMM; *F*_(1,30)_ = 12.572, *P*=0.001; Figure 2B). This identifies an acute transcriptional response of the yolk sac to mIA, as well as a differential temporality of response between placental and yolk sac tissues. Further, we found a significant interaction between treatment and sex (GLMM; *F*_(1,30)_ = 8.22, *P*=0.008), with the significant difference being driven by a higher expression of yolk sac *Tlr3* in female fetuses in the poly(I:C) treatment group compared with males at 3 h post-treatment.

However, at GD16, 24 h post-treatment, there was a significantly increased placental expression of *Tlr3* (GLMM; *F*_(1,26)_ = 6.09, *P*=0.020) in the poly(I:C) group compared with vehicle control (Figure 2A). Fetal sex was a significant predictor of placental *Tlr3* expression (GLMM; *F*_(1,26)_ = 6.30, *P*=0.019). The interaction between treatment and sex was also significant (GLMM; *F*_(1,26)_ = 11.59, *P*=0.002) with males in the poly(I:C) treatment group having a higher placental *Tlr3* expression than females. In contrast, yolk sac *Tlr3* expression at this gestational stage was more marginally affected, with a non-significant trend towards an increase (GLMM; *F*_(1,25)_ = 3.28, *P*=0.082). At GD21, 6 days following treatment, *Tlr3* gene expression did not differ between treatment groups in the placenta (GLMM; *F*_(1,29)_ = 0.085, *P*=0.773; Figure 2A), nor was there any impact of fetal sex (GLMM; *F*_(1,29)_ = 0.012, *P*=0.912). Likewise, in yolk sac at GD21, *Tlr3* gene expression was not significantly different between treatment groups (GLMM; *F*_(1,31)_ = 0.547, *P*=0.465; Figure 2B).

### Poly(I:C) up-regulates gene expression of pro-inflammatory cytokines in the placenta and yolk sac

**Figure 3:**
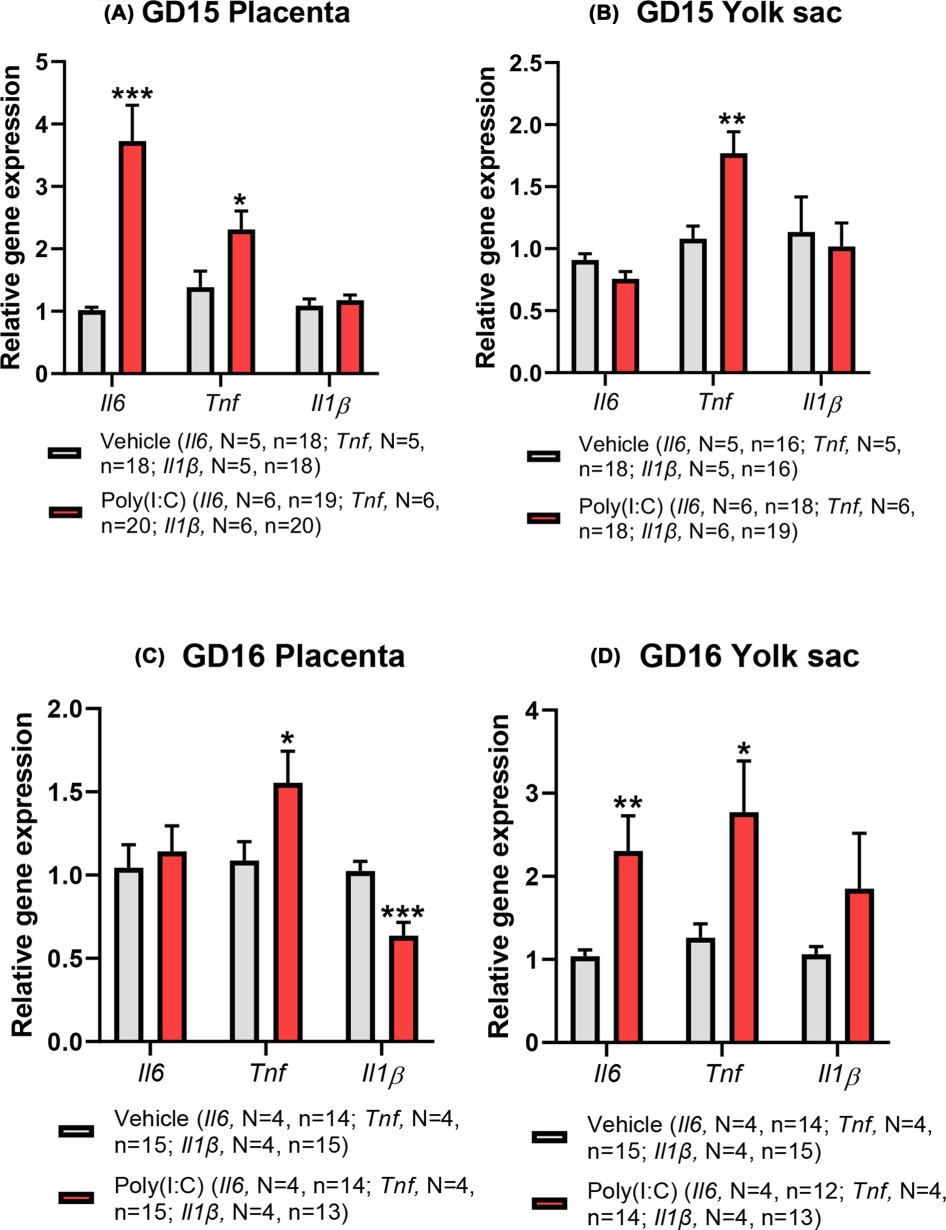
Effect of poly(I:C) treatment on mRNA expression of pro-inflammatory cytokines in fetus-matched placenta (A, C) and yolk sac (B, D) at GD15 and GD16, respectively Both tissues exhibit up-regulated cytokine transcription following poly(I:C) treatment with effects that are tissue-, time- and cytokine-dependent. Data presented as mean + SEM. *P<0.05, **P<0.01, ***P<0.001 vs vehicle control.

Having confirmed acute transcriptional changes in both placenta and yolk sac in response to mIA (Figure 2A,B), we next investigated the effect of poly(I:C) treatment on transcript levels of genes encoding IL-6, TNFα and IL-1β, mapping the tissue expression of these genes at GD15 and GD16, over the same temporal window of raised maternal pro-inflammatory cytokine plasma concentrations (Figure 1). At GD15, 3 h following poly(I:C) administration, expression of *Il6* was significantly increased in the placenta (GLMM; *F*_(1,33)_ = 20.68, *P*<0.001; Figure 3A), but this increase in placental *Il6* expression did not persist 24 h after treatment (GLMM; *F*_(1,24)_ = 0.21, *P*<0.653; Figure 3C). In contrast, in the yolk sac at GD15 (Figure 3B), treatment with poly(I:C) showed a trend approaching significance towards decreased *Il6* expression (GLMM; *F*_(1,30)_ = 4.00, *P*=0.055). Interestingly, however, at 24 h post-poly(I:C) treatment, yolk sac expression of *Il6* was significantly raised in the poly(I:C) group (GLMM; *F*_(1,22)_ = 12.06, *P*=0.002; Figure 3D), contrasting with the normalised placental *Il6* expression at this stage.

*Tnf* mRNA expression was also significantly elevated at GD15 by poly(I:C) treatment in both the placenta (GLMM; *F*_(1,34)_ = 5.03, *P*=0.032; Figure 3A) and yolk sac (GLMM; *F*_(1,32)_ = 11.58, *P*=0.002; Figure 3B), and remained significantly raised in both the placenta (GLMM; *F*_(1,26)_ = 5.27, *P*=0.030; Figure 3C) and yolk sac (GLMM; *F*_(1,25)_ = 5.25, *P*=0.031; Figure 3D) at GD16 compared with vehicle control.

These trends contrast with *Il1β* expression, which was not significantly affected by poly(I:C) treatment at GD15 in either the placenta (GLMM; *F*_(1,34)_ = 0.43, *P*=0.519; Figure 3A) or yolk sac (GLMM; *F*_(1,31)_ = 0.13, *P*=0.722; Figure 3B). This lack of effect persisted in the yolk sac at GD16, 24 h after poly(I:C) treatment (Figure 3D), whilst, in contrast, placental *Il1β* gene expression was significantly reduced at GD16 (GLMM; *F*_(1,24)_ = 18.83, *P*<0.001; Figure 3C).

These data show that poly(I:C)-induced mIA alters the transcriptional regulation of genes encoding pro-inflammatory cytokines in the placenta and yolk sac in a tissue-, cytokine- and time-dependent manner.

### Maternal plasma BCAA concentration is reduced, but placental BCAA concentration is increased, 24 h after poly(I:C) treatment

**Figure 4:**
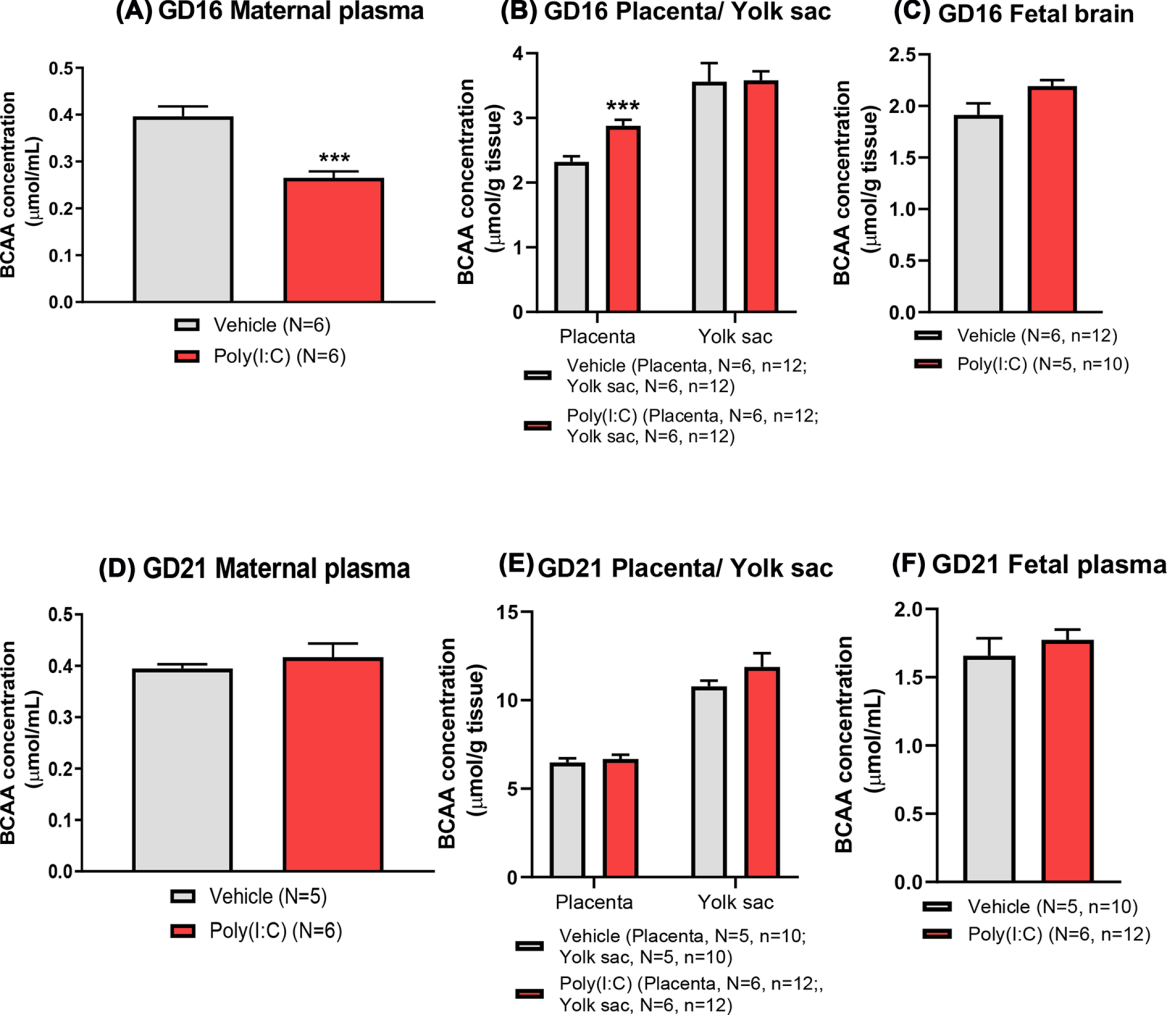
The concentration of BCAAs in the maternal plasma, placental/yolk sac and fetal tissues at GD16 (A–C; 24 h) and GD21 (D–F; 6 days) following poly(I:C) administration At GD16 (**A–C**), poly (I:C) treatment caused a significant reduction in maternal plasma BCAA concentration, contrasting with the increase in placental tissue BCAA concentration whereas there was no change in yolk sac with a non-significant trend (*P*=0.061) towards increased BCAA concentration in fetal brain. At GD21 (**D–F**), maternal plasma and fetal tissue BCAA concentrations were not significantly different between treatment groups. Data presented as mean + SEM. ***P<0.001 vs vehicle control.

Poly(I:C) administration to pregnant dams on GD15 caused a significant decrease in maternal plasma BCAA concentration 24 h after treatment on GD16 (GLM; *F*_(1,10)_ = 27.09, *P*<0.001; Figure 4A). In contrast, at GD21, 6 days following treatment, maternal plasma BCAA concentration was normalised to control concentration (Figure 4D). Against this background of reduced maternal BCAA concentration at GD16, placental BCAA concentration was increased (GLMM; *F*_(1,20)_ = 16.98, *P*=0.001), although no effect was observed in the yolk sac (Figure 4B). At GD16, it was not possible to harvest fetal blood, so BCAA concentration in the fetal brain was measured as an alternative fetal tissue of relevance to NDDs, to obtain an index of fetal compartment BCAA concentration. Poly(I:C) treatment induced a non-significant trend towards increased BCAA concentration in fetal brain (GLMM; *F*_(1,18)_ = 3.98, *P*=0.061; Figure 4C). At GD21, there was no significant difference in placental or yolk sac BCAA concentration between groups (Figure 4E) or in fetal-matched plasma (Figure 4F). However, as expected [50], fetal plasma BCAA concentration was significantly higher than maternal plasma BCAA at GD21 regardless of treatment group (GLMM; *F*_(1,22)_ = 138.87; *P*<0.001), demonstrating that the placenta was able to maintain the concentrative transport of amino acids towards the fetus in both control and poly(I:C) groups. Further, in keeping with the rise in rat fetal BCAA accumulation as gestation progresses towards term [51], tissue BCAA concentrations were significantly higher at GD21 compared with GD16 in both placenta (GLMM; *F*_(1,42)_ = 492.15; *P*<0.001) and yolk sac (GLMM; *F*_(1,42)_ = 286.41; *P*<0.001) regardless of treatment group (GLMM; *F*_(1,84)_ = 568.28; *P*<0.001).

### Poly(I:C) affects system L amino acid transporter subtype expression differentially in the placenta and yolk sac at GD21

**Figure 5:**
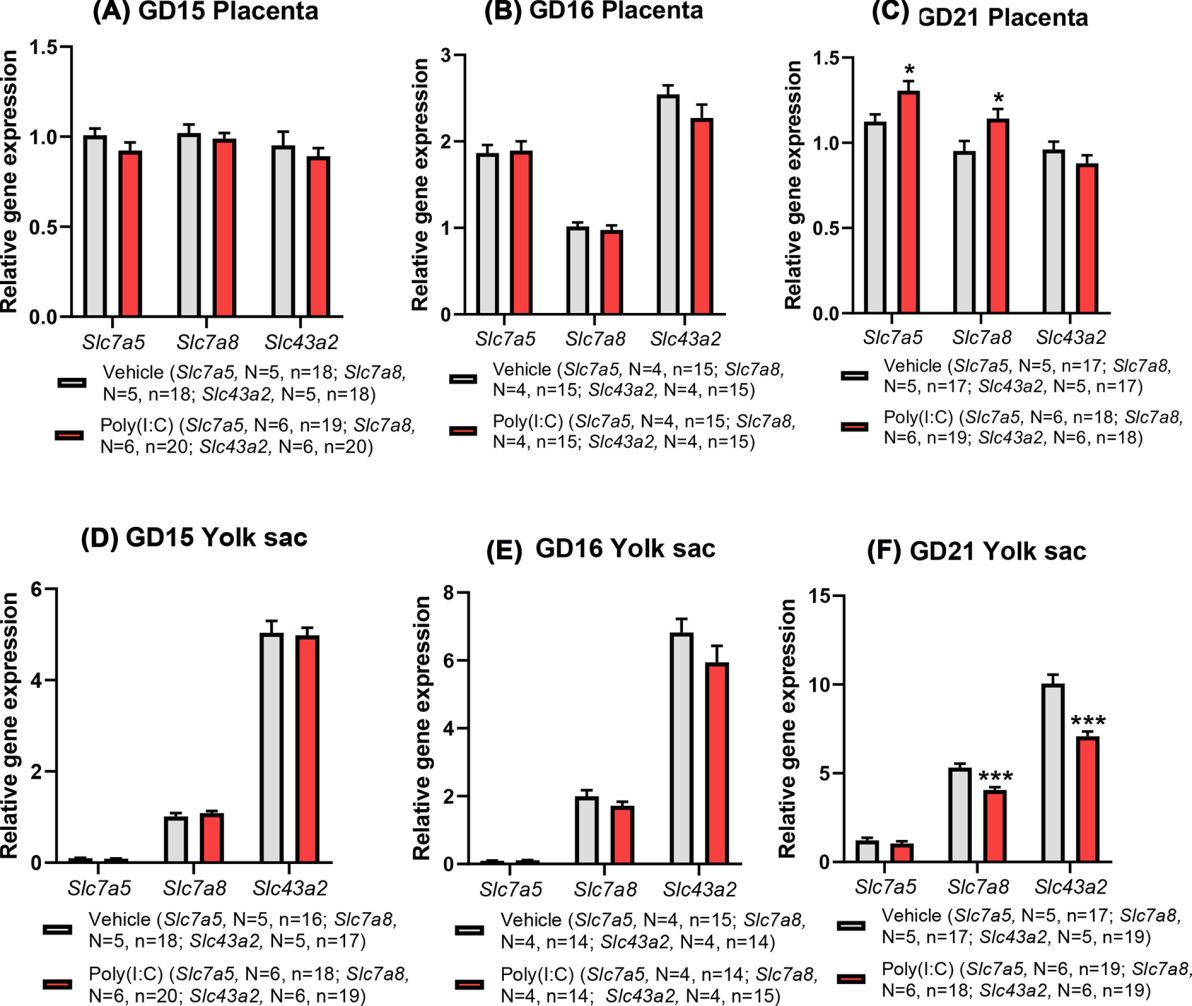
Gene expression of system L transporter subtypes in placenta (A–C) and yolk sac (D–F) at GD15 (3 h), GD16 (24 h) and GD21 (6 days) following poly(I:C) treatment Only at GD21 were differences observed in system L subtype gene expression between treatment groups in both placenta and yolk sac. Data presented as mean + SEM. *P<0.05, ***P<0.001 vs vehicle control.

Placental and yolk sac gene expression of all three LAT-encoding genes was demonstrated at each gestational age (Figure 5A–F). LAT protein expression studies were focused on GD16 (24 h post-treatment), as this was accompanied by a reduced maternal BCAA concentration (Figure 4A), increased placental BCAA concentration (Figure 4B) and altered expression of genes encoding pro-inflammatory cytokines in both tissues (Figure 3). Whilst LAT1, LAT2 and LAT4 were readily detectable at the protein level in placental lysates at GD16 (Supplementary Figure S4A), LAT1 was undetectable in yolk sac, in striking contrast with LAT2 and LAT4 which were both highly expressed (Supplementary Figure S4B), in agreement with our previous observations [32]. It is interesting to note that when maternal BCAA concentration was reduced at GD16 in response to poly(I:C) treatment (Figure 4A), there was no impact on placental or yolk sac system L subtype gene expression (Figure 5B,E); transcriptional changes in expression were only observed at GD21 (Figure 5C,F) when maternal plasma BCAA concentration was unaffected by treatment (Figure 4D). Further, placenta and yolk sac genes encoding each system L subtype were differentially affected by poly(I:C)-induced mIA at GD21 (Figure 5C,F), while also indicating that both tissues exhibited enduring transcriptional effects 6 days after poly(I:C) treatment.

Placental expression of *Slc7a5* showed a marginal non-significant trend overall by treatment on GD15 (GLMM; *F*_(1,33)_ = 3.21, *P*=0.082; Figure 5A). However, the interaction between treatment and sex was significant indicating a decrease in expression in the poly(I:C) group specific to males only (GLMM; *F*_(1,33)_ = 4.23, *P*=0.048). There was no significant difference in placental *Slc7a5* expression at GD16 (GLMM; *F*_(1,26)_ = 0.053, *P*=0.82; Figure 5B), and neither was placental LAT1 expression affected by treatment (GLMM; *F*_(1,19)_ = 0.073, *P*=0.79; Supplementary Figure S4A). In contrast, at GD21, placental expression of *Slc7a5* was significantly increased in the poly(I:C) group (GLMM; *F*_(1,31)_ = 6.52, *P*=0.016; Figure 5C). For yolk sac, no significant changes in *Slc7a5* expression were associated with poly(I:C) treatment on GD15 (GLMM; *F*_(1,30)_ = 1.51, *P*=0.23; Figure 5D), GD16 (GLMM; *F*_(1,25)_ = 1.57, *P*=0.22; Figure 5E) or GD21 (GLMM; *F*_(1,32)_ = 0.77, *P*=0.39; Figure 5F).

There was no overall significant effect of treatment on placental *Slc7a8* expression at GD15 (GLMM; *F*_(1,34)_ = 0.97, *P*=0.332; Figure 5A). However, the interaction between treatment and sex was once again significant (GLMM; *F*_(1,34)_ = 6.32, *P*=0.017), revealing a significant effect of reduced *Slc7a8* expression in the female group. At GD16, there were no differences between treatment groups with respect to placental *Slc7a8* gene expression (GLMM; *F*_(1,26)_ = 0.295, *P*=0.592; Figure 5B). However, there was a non-significant trend towards reduced expression of placental LAT2 protein (GLMM; *F*_(1,20)_ = 3.60, *P*=0.072, Supplementary Figure S4A). In contrast, at GD21, placental expression of *Slc7a8* was increased in the poly(I:C) group (GLMM; *F*_(1,32)_ = 5.44, *P*=0.026; Figure 5C), in common with the trend seen for *Slc7a5*. In the yolk sac, *Slc7a8* expression was not affected by treatment with poly(I:C) at GD15 and GD16 (Figure 5D,E), and LAT2 protein expression was also unaffected by treatment at GD16 (GLMM; *F*_(1,16)_ = 0.389, *P*=0.54; Supplementary Figure S4B). On the other hand, yolk sac *Slc7a8* expression at GD21 was significantly reduced by poly(I:C) as compared to vehicle control (GLMM; *F*_(1,31)_ = 21.55, *P*<0.001; Figure 5F), which was in the opposing direction observed in fetus-matched placental tissue.

Placental *Slc43a2* was unaffected by poly(I:C) administration at GD15 (GLMM; *F*_(1,34)_ = 0.61, *P*=0.44; Figure 5A), GD16 (GLMM; *F*_(1,26)_ = 1.90, *P*=0.18; Figure 5B) and GD21 (GLMM; *F*_(1,31)_ = 1.95, *P*=0.17; Figure 5C). Similarly, placental LAT4 protein expression was also unaffected by treatment at GD16 (GLMM; *F*_(1,20)_ = 0.017, *P*=0.90; Supplementary Figure S4A). *Slc43a2* expression was also not altered by poly(I:C) treatment in the yolk sac at GD15 (GLMM; *F*_(1,32)_ = 0.036, *P*=0.85; Figure 5D) and GD16 (GLMM; *F*_(1,25)_ = 2.02, *P*=0.17; Figure 5E). Yolk sac LAT4 protein expression was also unaffected by treatment at GD16 (GLMM; *F*_(1,17)_ = 2.046, *P*=0.17; Supplementary Figure S4B). However, in contrast, there was a significant reduction effect of poly(I:C) treatment on *Slc43a2* expression in the yolk sac at GD21 (GLMM; *F*_(1,32)_ = 28.24, *P*<0.001; Figure 5F), contrasting with the lack of effect in placenta.

### Maternofetal ^14^C-leucine transport shows a time-dependent response to poly(I:C)-induced mIA

**Figure 6:**
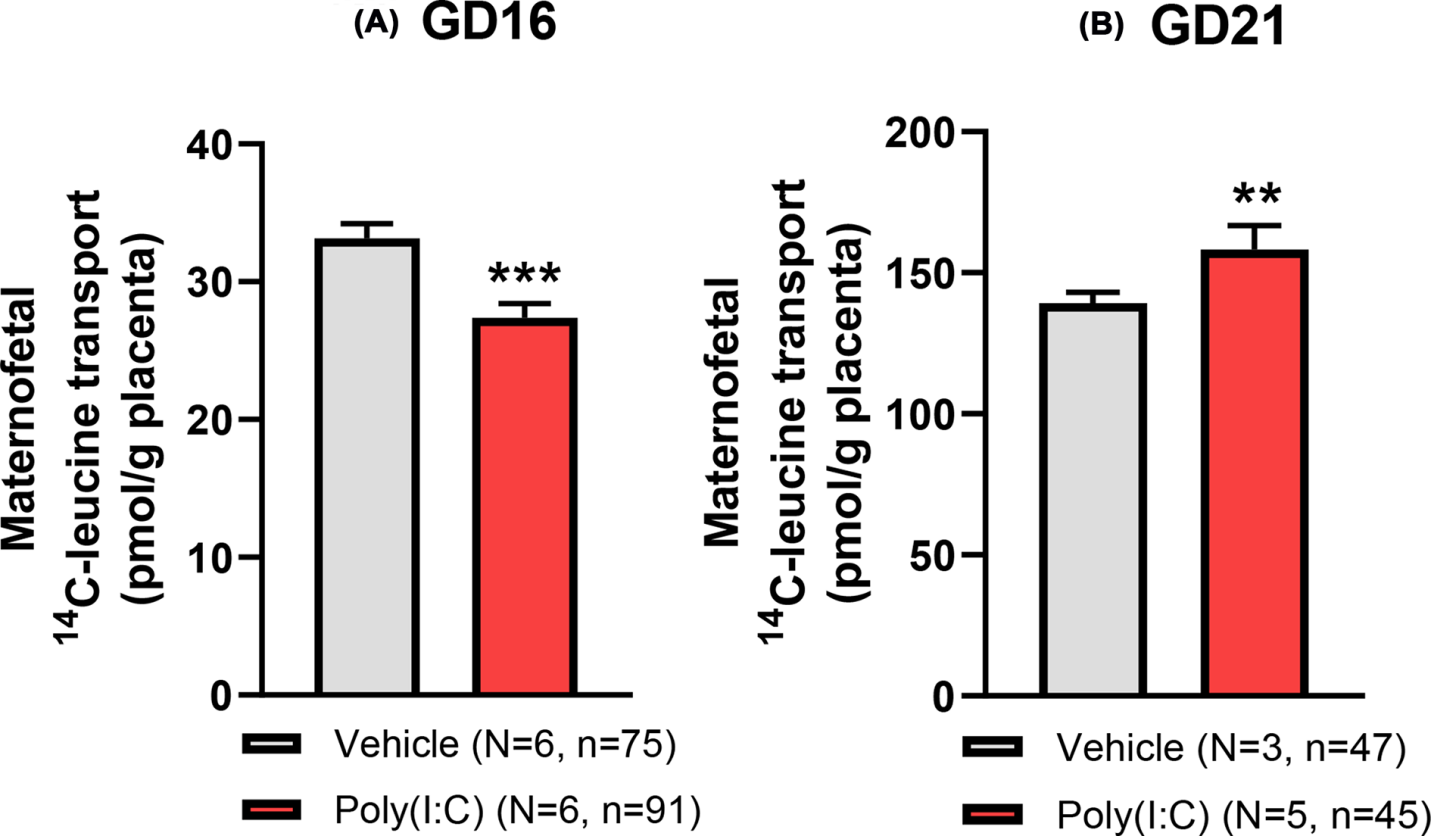
The effect of poly(I:C) treatment on the maternofetal transport of ^14^C-leucine at (A) GD16 (24 h) and (B) GD21 (6 days) following treatment Maternofetal transport of ^14^C-leucine demonstrated a biphasic response to poly(I:C)-induced mIA, with reduced transport at GD16 contrasting with the increased transport at GD21 compared with vehicle control. Data presented as mean + SEM. **P<0.01, ***P<0.001 vs vehicle control.

The maternofetal transport of ^14^C-leucine showed a striking increase from GD16 to GD21 with the relative rate of ^14^C-leucine transport increasing approximately 4-fold over this late gestational period (GLMM; *F*_(1,260)_ = 945.53, *P*<0.001), with this gestation-dependent increase observed for both groups (Figure 6A,B). Such a gestational increase in placental system L transporter-mediated activity accords with the increased amino acid requirement to support fetal growth towards term [51], with the exponential rise in fetal weight amounting to ∼14-fold increase over this period (Table 1). There was, however, an effect of poly(I:C) treatment. At GD16, 24 h after treatment with poly(I:C), fetal accumulation of ^14^C-leucine was significantly reduced (GLMM; *F*_(1,164)_ = 50.92, *P*<0.001; Supplementary Figure S5A), also observed in the placenta (GLMM; *F*_(1,162)_ = 8.14, *P*=0.005; Supplementary Figure S5B), and interestingly, also in the yolk sac (GLMM; *F*_(1,165)_ = 29.69, *P*<0.001; Supplementary Figure S5C), demonstrating that fetal delivery of maternal ^14^C-leucine and tissue accumulation of tracer was diminished. Consistent with this, maternofetal transport of ^14^C-leucine across the placenta was significantly reduced at GD16 (GLMM; *F*_(1,162)_ = 15.87, *P*<0.001; Figure 6A). Additionally, the interaction between treatment and sex was significant, showing that decreased ^14^C-leucine accumulation was more pronounced in the placenta (GLMM; *F*_(1,162)_ = 6.24, *P*=0.013) and yolk sac of female fetuses (GLMM; *F*_(1,165)_ = 6.13, *P*=0.014) at GD16.

In contrast, at GD21, the pattern was reversed. Six days after poly(I:C) treatment, we found a significant increase in fetal (GLMM; *F*_(1,95)_ = 9.85, *P*=0.002; Supplementary Figure S5D), placental (GLMM; *F*_(1,95)_ = 17.57, *P*<0.002; Supplementary Figure S5E) and yolk sac (GLMM; *F*_(1,95)_ = 6.20, *P*=0.015; Supplementary Figure S5F) accumulation of ^14^C-leucine, demonstrating an enhancement in the fetal delivery and tissue accumulation of ^14^C-leucine. Thus, maternofetal transport of ^14^C-leucine across the placenta was significantly increased at GD21 (GLMM; *F*_(1,95)_ = 8.94, *P*=0.004; Figure 6B). Together these data demonstrate a biphasic response of placental system L activity to poly(I:C)-induced mIA, with an initial acute reduction in placental ^14^C-leucine transport observed 24 h after treatment at GD16, followed by an adaptive up-regulation 6 days after treatment at GD21.

## Discussion

This study sought to address significant gaps in our understanding of how maternal infection leads to altered placental and yolk sac transport activity and fetal development through impacts on placental- and yolk sac-elicited effects. First, we determined acute responses to mIA at both 3 h and 24 h post-poly(I:C) treatment on GD15 and GD16, respectively, as well as longer-term physiological responses at GD21, 6 days following poly(I:C) treatment. This allowed assessment over a period of neurodevelopment likely to be vulnerable to the impacts of mIA caused by prenatal maternal infection. Second, we examined concurrent responses to mIA in both sexes in the maternal and fetoplacental compartments, including concomitant effects on placenta and yolk sac, which provide essential amino acids to the developing fetal brain.

Our demonstration of *Tlr3* gene expression (encoding the endosomal TLR3 receptor) in both rat placenta and yolk sac implies that both tissues have the potential to mediate responses to poly(I:C) through the TLR3 signalling cascade leading to the activation of mitogen-activated protein kinases (MAPK) and nuclear factor-κB (NF-κB) to stimulate inflammatory cytokine gene activity and production [40,49], with downstream effects such as altered fetal cortical neurogenesis, an effect abrogated in TLR3-deficient mice [52].

In agreement with previous studies [47,48], we have demonstrated that poly(I:C)-induced mIA stimulated placental *Tlr3* gene expression at 24 h post-treatment. By contrast, we observed up-regulated yolk sac *Tlr3* expression as early as 3 h post-treatment, suggesting a temporal difference in *Tlr3* transcriptional response between these two fetal tissues.

### mIA effect on maternal cytokines

We confirmed the successful induction of mIA by poly(I:C) treatment by the significant elevation in maternal pro-inflammatory cytokines IL-6 and TNFα at 2 and 3 h post-poly(I:C) injection respectively. By 24 h, however, these cytokine responses had declined to baseline, in broad agreement with other acute pro-inflammatory cytokine responses using the same form and source of poly(I:C) in pregnant mice [39,53]. In contrast with previous studies in pregnant mice [39], we did not observe a contemporaneous change in maternal plasma IL-1β. These differences in cytokine responses may reflect different species, strain, gestational timing, dose, route and source of poly(I:C) administration [20,39,41,54]. We thus advocate, like others, much more rigorous reporting of mIA model experimental conditions together with the inclusion of maternal immunogenic responses as a validation of the elicited mIA response [41,42].

### mIA effect on fetal body and brain weight

The induction of mIA in pregnant rat dams using LMW poly(I:C) on GD15 did not compromise pregnancy progression and fetal weight was not altered at any gestational age examined by poly(I:C) treatment, with fetuses from both groups displaying the expected gestational weight gain with male fetuses heavier than females [25,55]. These data also accord well with reports of unaltered rat pup bodyweight at birth (postnatal day 1) following prenatal mIA induction by poly(I:C) [56]. Importantly, both brain weight (as a proxy of brain growth) and brain:bodyweight ratio were significantly increased at all gestational ages, whereas we found no change in fetal liver weight at any gestational age. This suggests fetal brain development may have been affected more profoundly by poly(I:C) treatment relative to other fetal organs.

The persistence of increased fetal brain mass in the poly(I:C)-treated group at all gestational ages implies that there were rapid and enduring changes in fetal brain developmental trajectory in response to mIA, even after the acute maternal-evoked pro-inflammatory cytokine responses had subsided. Poly(I:C) rodent models have demonstrated acute dose-dependent increases in fetal brain cytokines with gestation-dependent differences, which may persist in specific brain regions in later life stages [18]. Such pro-inflammatory cytokine responses have been postulated to disrupt the normal cytokine balance that underpins the modulation of fetal brain development with respect to neuronal proliferation and differentiation, synaptic plasticity, neural transmission and signalling [18,57], resulting in an altered neurodevelopmental trajectory with divergent postnatal dysfunctional behavioural phenotypes [54]. Indeed, previous studies in rats have shown that poly(I:C) treatment results in a down-regulation of transcripts associated with neurodevelopment [18], stimulated neural proliferation, and an increased cortical thickness of the fetal brain [37,58].

It is notable that we observed a change in fetal brain weight as early as 3 h post-poly(I:C) treatment, when maternal cytokine concentrations were elevated and a timeframe concomitant with significantly raised *Il6* mRNA and IL-6 protein in fetal brain [18,39,59,60], with similar trends seen for TNFα [18,39]. This confirms rapid mIA-dependent cytokine changes in the fetal compartment. It is also worth commenting that a single transient rise in circulating maternal IL-6 concentration is sufficient to induce a very rapid increase in the proliferation of forebrain neural precursors and the number of embryonic cortical cells, and that this intrinsic alteration in cortical development can persist into postnatal life [61]. Hence, morphological changes in cortical development may account for our observation of a heavier fetal brain weight in the poly(I:C)-challenged fetuses at all measured gestational stages.

### mIA effect on placenta and yolk sac cytokine expression

Consistent with the notion of rapid cytokine changes in the fetal compartment, up-regulated *Il6* gene expression was seen in the placenta at 3 h post-poly(I:C) treatment, whereas this was not observed in the yolk sac until 24 h later. Yet, both tissues elicited an increase in *Tnf* gene expression at 3 and 24 h post-poly(I:C) administration. This rapid placental response agrees well with previous reports of increased placental *Il6* and *Tnf* mRNA expression at 3 h [59,62] or 6 h [63] post-poly(I:C) treatment. Likewise, intravenously administered poly(I:C) in pregnant mice also showed increased yolk sac gene expression of *Il6* and *Tnf* after 24 h following treatment [64], but in contrast, we did not observe a parallel increase in *Il1β* gene expression. Taken together, our study has shown an acute induction of inflammatory cytokine transcription in placenta and yolk sac, which exhibits temporal dependence and is cytokine-specific.

Transcriptional cytokine changes are associated with increased placental IL-6 [53,62] and TNFα [65] protein concentration, which itself can induce IL-6 synthesis [66], with rodent placental trophoblast cells able to synthesise a variety of pro-inflammatory cytokines in response to poly(I:C) via TL3-dependent mechanisms [53]. However, the mIA-induced increase in placental IL-6 concentration may reflect synthesis by activated maternal decidual leucocytes [62]. The neutralisation of IL-6 activity and ablation of *Il6* gene activity during fetal development can prevent the behavioural deficits caused by poly(I:C)-induced mIA [67]. Hence, maternal IL-6 is postulated to be a key mediator in the mechanisms underpinning the mIA-elicited changes to fetal brain development and the enduring behavioural deficits reminiscent of NDDs such as SZ in the offspring [67]. These rodent studies imply that maternal IL-6 crosses the placenta and enters the fetal circulation [68] and then the fetal blood–brain barrier [69] to elicit effects directly on the fetal brain through various signalling pathways [67]. Alternatively, mIA-evoked responses could influence placental and yolk sac cytokine synthesis [53,62,64] and function with downstream impacts on fetal brain developmental trajectory and function.

### mIA effect on placental and fetal weight

The positive correlation between placental and fetal weights, demonstrated in our study, and others [55], and in common with other species [46], signifies the importance of appropriate placental development and function to fetal growth. Poly(I:C) treatment reduced placental weight 24 h following treatment, accompanied by a diminished placental area, with a trend towards a reduced placental weight at GD21, in agreement with our previous observations [20,25] and accompanied by an increased fetal:placental weight ratio at both gestational stages. However, these changes were not associated with a significant change in fetal body weight (in line with our previous observations [20,25]), and did not impair the normal exponential rise in rat fetal weight that occurs over late gestation [51]. These observations led us to speculate that both the sustained increase in fetal:placental weight ratio, as a proxy of placental transport efficiency [46], and the raised brain:fetal body weight ratio following poly(I:C) treatment were underpinned by an altered placental provision of amino acids to the developing fetus, as precursors for protein synthesis and accretion. As gestation advances towards term in rat pregnancy, there is a high fetal demand for essential amino acids, as exemplified by the progressive gestational increase in the fetal accumulation of these amino acids [51]. This accumulation is likely be contributed to by system-L mediated transport across both the rat placenta and yolk sac [32]. Such a concept is supported by our evidence of increased placental and yolk sac BCAA tissue concentrations as gestation advanced, as well as the raised maternofetal leucine transport capacity at GD21, relative to that at GD16.

### mIA effect on BCAA and system L subtype expression

Our initial observation that poly(I:C)-induced mIA reduced maternal BCAA concentration agrees well with previous studies in pregnant rats [63]. A decrease in leucine and isoleucine concentration in fetal brain 24 h post-treatment has been reported whereas other system L substrates such as tryptophan were increased at 48 h [63]. In contrast, we found a trend to increased BCAA concentration in fetal brain at 24 h following poly(I:C) treatment which mirrored the contemporaneous increase in placental BCAA concentration. Whilst these differences may be attributable to the different source of poly(I:C) used previously (Sigma-Aldrich) [63], it should be noted that others report acute increases in leucine, isoleucine and valine in the fetal brains of LPS-treated mice with increased BCAA metabolites also found in the amniotic fluid [70]. However, by GD21, maternal BCAA concentration had normalised with no effect of poly(I:C) treatment on BCAA concentration in placenta, yolk sac or fetal plasma, highlighting the acute nature of the effect on BCAA homeostasis.

This prompted us to examine whether the fetal provision of leucine and expression of placental and yolk sac system L subtypes were altered acutely (at 3 h (GD15) or 24 h (GD16)) or more chronically (at six days (GD21)) post-poly(I:C) treatment and whether induction of mIA impacted on placental system L transport capacity.

In agreement with our previous study at GD21 [32], expression of *Slc7a5, Slc7a8* and *Slc43a2* genes encoding LAT1, LAT2 and LAT4 subtypes was confirmed in rat placenta at all gestational ages examined, with yolk sac expression of these transcripts also confirmed with a relatively low expression of *Slc7a5.* Similar to others [63], we found no effect on placental and yolk sac *Slc7a5, Slc7a8* or *Slc43a2* mRNA expression 24 h following poly(I:C) treatment in pregnant rat dams. However, 6 days after poly(I:C) treatment (GD21), both *Slc7a5* and *Slc7a8* were up-regulated in placenta, whilst *Slc7a8* and *Slc43a2* were down-regulated in yolk sac. Taken together, these observations indicate that there is differential, tissue- and temporal-dependence of transcriptional regulation of transporter gene expression in fetal tissues in response to poly(I:C)-induced mIA, and that such effects are apparent several days after the induction of mIA and peak maternal cytokine response, in broad agreement with the observation of others [63].

We did not determine whether the placental BCAA increase 24 h following poly(I:C) treatment was attributable to an altered BCAA concentration in either the junctional zone (JZ), labyrinth zone (LZ) or both zones of the placenta. Both these placental zones express LAT1, LAT2 and LAT4, although there is a relatively higher expression of LAT1 and LAT4 in the LZ, the part of the placenta vascularised by fetal capillaries to enable maternofetal exchange, contrasting with LAT2 dominance in the JZ, which has a primary endocrine function [32]. In the yolk sac, LAT2 and LAT4 expression dominate [32], but yolk sac LAT2 and LAT4 protein expression was unaltered 24 h following poly(I:C) treatment with no effect on yolk sac BCAA concentration, suggesting differential responses between these fetal tissues. Whether this reflects differences in the relative expression of LAT subtypes between the tissues or the effect of local regulatory factors on system L activity is not clear.

### Maternofetal transport of ^14^C-leucine

The higher placental BCAA concentration 24 h after poly(I:C) treatment would be consistent with a stimulated uptake of amino acids by system L into the placenta, or alternatively a diminished efflux of amino acids to the fetus. Our measurement of maternofetal transport of ^14^C-leucine, a model substrate for system L [27] and the most abundant BCAA in proteins [71], clearly demonstrated reduced maternofetal transport of ^14^C-leucine 24 h after poly(I:C) treatment. By contrast, at GD21, we found the opposing effect, with maternofetal transport of ^14^C-leucine increased by poly(I:C) treatment. We interpret this biphasic response of placental leucine transport to mIA as an initial impairment followed by an adaptive, compensatory up-regulation in placental transport capacity. Interestingly, this did not affect fetal body weight on GD16, suggesting the effect was either transitory in nature or rapidly compensated for by an up-regulation in amino acid transport to the fetus. Consistent with this notion, maternofetal transport of ^14^C-leucine was increased in the poly(I:C) group at GD21.

We cannot discount effects on other transport mechanisms such as system A amino acid transporter, as placental uptake of amino acids by system A provides amino acid substrates for exchange by system L-mediated activity [26,27]. For example, TNFα stimulates system A-mediated amino acid transporter activity [72,73] through a MAPK-dependent pathway [72] and IL-6 also increases system A activity through increased phosphorylation of signal transducer and activator of transcription 3 (STAT3) [73]. *Slc38a1* and *Slc38a2* mRNA (encoding SNAT1 and SNAT2 subtypes of system A) are up-regulated in rat placenta following poly(I:C)-induced mIA, although SNAT2 protein expression remains unchanged [63]. However, neither IL-6 nor TNFα at comparable doses were found to alter system L-mediated leucine uptake in the same cell type [73].

Both the JZ and LZ of rat placenta express *Il6, Tnf* and *Il1β* transcripts with higher expression of *Il6* in the LZ, whereas *Tnf* and *Il1β* have higher JZ expression, although at the protein level only IL-1β remains significantly different [74]. It is therefore plausible that functional activities of both the JZ and LZ could be affected by a perturbed cytokine environment. There may also be direct effects of cytokines on transporter activity within the LZ. Previous studies have shown that an acute injection of TNFα to pregnant rat dams on GD20, significantly reduced placental transport of the leucine analogue ^14^C-cycloleucine [75]. However, later studies using rat placental plasma membrane vesicles demonstrated that the maternal infusion of TNFα over 7 days in pregnant rats resulted in a significantly increased Na^+^-independent leucine uptake, which characterises system L activity [76]. This pattern of response mirrors the biphasic response seen in the present study in response to poly(I:C)-mediated mIA, and it is tempting to speculate that this may be modulated by placental cytokine environment and downstream signalling events, perhaps through epigenetically regulated mechanisms that may have enduring neurobehavioural consequences for the offspring [18].

### Proposed mechanism of mIA effect on fetal brain development

Therefore, our evidence supports a mechanistic framework whereby mIA, in concert with elevated maternal cytokine concentrations, alters acutely maternal BCAA homeostasis, placental BCAA concentration and transport of leucine to the fetus in a temporal-dependent manner post poly(I:C)-induction of mIA. We propose that after the induction of mIA, altered placental system L-mediated delivery of leucine to the fetal brain, essential for protein synthesis and neuromodulation of brain function [71], affects fetal brain amino acid homeostasis and function. In conjunction with transcriptional [18] and metabolomic [63,70] changes, this alters fetal neurodevelopment *in utero* predisposing the fetus to NDD risk in later life. Enduring effects on placental amino acid transport function may have functional ramifications for neurological function [71]. Indeed, rodent mIA offspring develop altered behavioural traits and impaired cognitive function of relevance to SZ including deficits in pre-pulse inhibition, novel object recognition and attentional shifting [5,77].

It is also noteworthy that system L, and each of the LAT1, LAT2 and LAT4 subtypes, transports methionine [27], the precursor for methylation, raising the possibility that fetal gene methylation events could be altered. Indeed, altered methylation of a number of genes has been documented in the offspring brain of poly(I:C)-treated dams [18].

In summary, our results demonstrate that prenatal infection, modelled here by poly(I:C)-induced mIA, influences physiological function in the maternal, placental and fetal compartments with a temporal dependency following the induction of mIA, as well as eliciting divergent transcriptional effects between placenta and yolk sac. Importantly, altered placental amino acid transport function and fetal provision of essential amino acids in response to mIA could impact on fetal neurodevelopmental pathways and influence risk of NDDs in later life, adding to our current understanding of the multiple pathways that converge to cause NDDs, whilst implicating a role for dysfunctional placental amino acid transport. In this context it is also worth emphasising that gene loci linked to SZ susceptibility risk are highly expressed in placenta [78]. A further novel aspect of this study is the demonstration that the yolk sac also elicits responses to mIA that are divergent to the placenta in some regards, but which, importantly, identify the rodent yolk sac as an immuno-inflammatory responsive tissue. This is highly relevant to the development of NDDs because the yolk sac is the source of microglia progenitors that migrate and colonise the embryonic brain [79,80]. Prenatal inflammation stimulates microglial activation, which is considered a risk factor for the development of NDDs such as SZ [80]. Further elucidation of mIA-invoked dysregulated placental and yolk sac functional pathways may provide an opportunity for the development of targeted interventions to ameliorate NDD risk.

## Clinical perspectives

- Studies investigating the effects of exposure to maternal infection in rodent models have focussed on offspring behavioural/cognitive changes while prenatal effects on placental and yolk sac amino acid transport function, crucial for normal fetal brain development, remain poorly defined.
- In a rat model of maternal immune activation, induced by the viral mimetic poly(I:C), we found reduced placental and yolk sac weights, increased fetal brain weight but unaltered fetal body weight, stimulated placental and yolk sac transcription for toll-like receptor 3 and pro-inflammatory cytokines but divergent transcriptional responses between tissues for genes encoding LAT amino acid transporters that transport essential amino acids, and an acute reduction in the maternofetal transport of ^14^C-leucine followed by compensatory increase with changes in branched-chain amino acids in maternal plasma and placenta.
- We identify a critical prenatal pathway linking maternal infection to altered offspring brain development and the subsequent potential for neurodevelopmental disease through dysregulated fetal amino acid provision.

## Supplementary Material

Supplementary Figures S1-S5 and Table S1

## Data Availability

Data are available from the corresponding author upon reasonable request.

## Abbreviations

BCAA: branched-chain amino acid
GD: gestational day
JZ: junctional zone
LAT: L-type amino acid transporter
LMW: low molecular weight
LPS: bacterial endotoxin lipopolysaccharide
LZ: labyrinth zone
mIA: maternal immune activation
NDD: neurodevelopmental disease
poly(I:C): polyinosinic:polycytidylic acid
SNAT: sodium-dependent neutral amino acid transporter
SZ: schizophrenia
TLR: toll-like receptor

## References

[1] Brown A.S. and Derkits E.J. (2010) Prenatal infection and schizophrenia: a review of epidemiologic and translational studies. Am. J. Psychiatry 167, 261–280 10.1176/appi.ajp.2009.09030361 20123911 PMC3652286

[2] Brown A.S. and Patterson P.H. (2011) Maternal infection and schizophrenia: implications for prevention. Schizophren. Bull. 37, 284–290 10.1093/schbul/sbq146 PMC3044639 21134972

[3] Brown A.S. (2012) Epidemiologic studies of exposure to prenatal infection and risk of schizophrenia and autism. Dev. Neurobiol. 72, 1272–1276 10.1002/dneu.22024 22488761 PMC3435457

[4] Saha S. et al. (2005) A systematic review of the prevalence of schizophrenia. PLoS Med. 2, e141 10.1371/journal.pmed.0020141 15916472 PMC1140952

[5] Knuesel I. et al. (2014) Maternal immune activation and abnormal brain development across CNS disorders. Nat. Rev. Neurol. 10, 643–660 10.1038/nrneurol.2014.187 25311587

[6] Patterson P.H. (2009) Immune involvement in schizophrenia and autism: etiology, pathology and animal models. Behav. Brain Res. 204, 313–321 10.1016/j.bbr.2008.12.016 19136031

[7] Meyer U. (2013) Developmental neuroinflammation and schizophrenia. Prog. Neuropsychopharmacol. Biol. Psychiatry 42, 20–34 10.1016/j.pnpbp.2011.11.003 22122877

[8] Feigenson K.A., Kusnecov A.W. and Silverstein S.M. (2014) Inflammation and the two-hit hypothesis of schizophrenia. Neurosci. Biobehav. Rev. 38, 72–93 10.1016/j.neubiorev.2013.11.006 24247023 PMC3896922

[9] Meyer U., Schwarz M.J. and Müller N. (2011) Inflammatory processes in schizophrenia: a promising neuroimmunological target for the treatment of negative/cognitive symptoms and beyond. Pharmacol. Ther. 132, 96–110 10.1016/j.pharmthera.2011.06.003 21704074

[10] Brown A.S. et al. (2004) Serologic evidence of prenatal influenza in the etiology of schizophrenia. Arch. Gen. Psychiatry 61, 774–780 10.1001/archpsyc.61.8.774 15289276

[11] Brown A.S. and Susser E.S. (2002) In utero infection and adult schizophrenia. Ment. Retard. Dev. Disabil. Res. Rev. 8, 51–57 10.1002/mrdd.10004 11921387

[12] Sarkar T., Patro N. and Patro I.K. (2019) Cumulative multiple early life hits - a potent threat leading to neurological disorders. Brain Res. Bull. 147, 58–68 10.1016/j.brainresbull.2019.02.005 30771410

[13] Fatemi S.H. and Folsom T.D. (2009) The neurodevelopmental hypothesis of schizophrenia, revisited. Schizophr. Bull. 35, 528–548 10.1093/schbul/sbn187 19223657 PMC2669580

[14] Fineberg A.M. and Ellman L.M. (2013) Inflammatory cytokines and neurological and neurocognitive alterations in the course of schizophrenia. Biol. Psychiatry 73, 951–966 10.1016/j.biopsych.2013.01.001 23414821 PMC3641168

[15] Macêdo D.S. et al. (2012) Animal models of prenatal immune challenge and their contribution to the study of schizophrenia: a systematic review. Braz. J. Med. Biol. Res. 45, 179–186 10.1590/S0100-879X2012007500031 22392187 PMC3854194

[16] Meyer U. and Feldon J. (2010) Epidemiology-driven neurodevelopmental animal models of schizophrenia. Prog. Neurobiol. 90, 285–326 10.1016/j.pneurobio.2009.10.018 19857543

[17] Bauman M.D. et al. (2014) Activation of the maternal immune system during pregnancy alters behavioral development of rhesus monkey offspring. Biol. Psychiatry 75, 332–341 10.1016/j.biopsych.2013.06.025 24011823 PMC6782053

[18] Woods R.M. et al. (2021) Maternal immune activation in rodent models: A systematic review of neurodevelopmental changes in gene expression and epigenetic modulation in the offspring brain. Neurosci. Biobehav. Rev. 129, 389–421 10.1016/j.neubiorev.2021.07.015 34280428

[19] Estes M.L. and McAllister A.K. (2016) Maternal immune activation: implications for neuropsychiatric disorders. Science 353, 772–777 10.1126/science.aag3194 27540164 PMC5650490

[20] Murray K.N. et al. (2019) Evolution of a maternal immune activation (mIA) model in rats: Early developmental effects. Brain Behav. Immun. 75, 48–59 10.1016/j.bbi.2018.09.005 30218784

[21] Forrest C.M. et al. (2012) Prenatal activation of toll-like receptors-3 by administration of the viral mimetic poly(I:C) changes synaptic proteins, N-methyl-D-aspartate receptors and neurogenesis markers in offspring. Mol. Brain 5, 22 10.1186/1756-6606-5-22 22681877 PMC3496691

[22] Wood T.C. et al. (2019) Mapping the impact of exposure to maternal immune activation on juvenile Wistar rat brain macro- and microstructure during early post-natal development. Brain *Neurosci. Adv.* 3, 2398212819883086 10.1177/2398212819883086 31742236 PMC6861131

[23] Vernon A.C. et al. (2015) Longitudinal in vivo maturational changes of metabolites in the prefrontal cortex of rats exposed to polyinosinic-polycytidylic acid in utero. Eur. Neuropsychopharmacol. 25, 2210–2220 10.1016/j.euroneuro.2015.09.022 26475576

[24] Wolff A.R., Cheyne K.R. and Bilkey D.K. (2011) Behavioural deficits associated with maternal immune activation in the rat model of schizophrenia. Brain Behav. Res. 225, 382–387 10.1016/j.bbr.2011.07.033 21816179

[25] Kowash H.M. et al. (2019) Poly(I:C) source, molecular weight and endotoxin contamination affect dam and prenatal outcomes, implications for models of maternal immune activation. Brain Behav. Immun. 82, 160–166 10.1016/j.bbi.2019.08.006 31415868

[26] Lager S. and Powell T.L. (2012) Regulation of nutrient transport across the placenta. J. Pregnancy 2012, 179827 10.1155/2012/179827 23304511 PMC3523549

[27] Cleal J.K. and Lewis R.M. (2008) The mechanisms and regulation of placental amino acid transport to the human foetus. J. Neuroendocrinol. 20, 419–426 10.1111/j.1365-2826.2008.01662.x 18266945

[28] Vaughan O.R. et al. (2017) Regulation of placental amino acid transport and fetal growth. Prog. Mol. Biol. Transl. Sci. 145, 217–251 10.1016/bs.pmbts.2016.12.008 28110752

[29] Rennie M.Y. et al. (2014) The Uteroplacental, Fetoplacental, and Yolk Sac Circulations in the Mouse. In The Guide to Investigation of Mouse Pregnancy(Croy B.A., Yamada A.T., DeMayo F.J. and Adamson S.L., eds), pp. 201–210, Academic Press, Boston 10.1016/B978-0-12-394445-0.00016-3

[30] Beckman D.A., Brent R.L. and Lloyd J.B. (1996) Sources of amino acids for organogenesis in the rat. 4. Protein synthesis during early mechanisms before envelopment of the embryo by the yolk sac. Placenta 17, 635–641 10.1016/S0143-4004(96)80082-8 8916213

[31] Beckman D.A. and Tu C. (1997) Leucine sources for 10.5 day rat conceptus in vivo. Reprod. Toxicol. 11, 875–877 10.1016/S0890-6238(97)00071-3 9407598

[32] Owaydhah W.H. et al. (2021) Differential expression of system L amino acid transporter subtypes in rat placenta and yolk sac. Placenta 103, 188–198 10.1016/j.placenta.2020.10.034 33160252

[33] Errasti-Murugarren E. and Palacín M.H. (2022) Heteromeric amino acid transporters in brain: from physiology to pathology. Neurochem. Res. 47, 23–36 10.1007/s11064-021-03261-w 33606172

[34] Sperringer J.E., Addington A. and Hutson S.M. (2017) Branched-chain amino acids and brain metabolism. Neurochem. Res. 42, 1697–1709 10.1007/s11064-017-2261-5 28417264

[35] Barke T.L. et al. (2019) Sex modifies placental gene expression in response to metabolic and inflammatory stress. Placenta 78, 1–9 10.1016/j.placenta.2019.02.008 30955704 PMC6461364

[36] Bale T.L. (2016) The placenta and neurodevelopment: sex differences in prenatal vulnerability. Dialogues Clin. Neurosci. 18, 459–464 10.31887/DCNS.2016.18.4/tbale 28179817 PMC5286731

[37] Baines K.J. et al. (2020) Maternal immune activation alters fetal brain development and enhances proliferation of neural precursor cells in rats. Front. Immunol. 11, 1145 10.3389/fimmu.2020.01145 32582210 PMC7295982

[38] Sauvageot C.M. and Stiles C.D. (2002) Molecular mechanisms controlling cortical gliogenesis. Curr. Opin. Neurobiol. 12, 244–249 10.1016/S0959-4388(02)00322-7 12049929

[39] Mueller F.S. et al. (2019) Influence of poly(I:C) variability on thermoregulation, immune responses and pregnancy outcomes in mouse models of maternal immune activation. Brain Behav. Immun. 80, 406–418 10.1016/j.bbi.2019.04.019 30980948

[40] Kawasaki T. and Kawai T. (2014) Toll-like receptor signaling pathways. Front. Immunol. 5, 461 10.3389/fimmu.2014.00461 25309543 PMC4174766

[41] Careaga M. et al. (2018) Variability in polyIC induced immune response: Implications for preclinical maternal immune activation models. J. Neuroimmunol. 323, 87–93 10.1016/j.jneuroim.2018.06.014 30196839 PMC6782055

[42] Kentner A.C. et al. (2019) Maternal immune activation: reporting guidelines to improve the rigor, reproducibility, and transparency of the model. Neuropsychopharmacology 44, 245–258 10.1038/s41386-018-0185-7 30188509 PMC6300528

[43] Jansson N. et al. (2006) Down-regulation of placental transport of amino acids precedes the development of intrauterine growth restriction in rats fed a low protein diet. J. Physiol. 576, 935–946 16916910 10.1113/jphysiol.2006.116509 PMC1892642

[44] Kalisch-Smith J.I. et al. (2017) Sex differences in rat placental development: from pre-implantation to late gestation. Biol. Sex Differ. 8, 17 10.1186/s13293-017-0138-6 28523122 PMC5434533

[45] Cohen E., Baerts W. and van Bel F. (2015) Brain-sparing in intrauterine growth restriction: considerations for the neonatologist. Neonatology 108, 269–276 10.1159/000438451 26330337

[46] Hayward C.E. et al. (2016) Placental adaptation: what can we learn from birthweight:placental weight ratio? Front. Physiol. 7, 28 10.3389/fphys.2016.00028 26903878 PMC4742558

[47] Chatterjee P. et al. (2012) Placental Toll-like receptor 3 and Toll-like receptor 7/8 activation contributes to preeclampsia in humans and mice. PLoS ONE 7, e41884 10.1371/journal.pone.0041884 22848646 PMC3407075

[48] Tinsley J.H. et al. (2009) Toll-like receptor 3 activation during pregnancy elicits preeclampsia-like symptoms in rats. Am. J. Hypertens. 22, 1314–1319 10.1038/ajh.2009.185 19779466

[49] Alexopoulou L. et al. (2001) Recognition of double-stranded RNA and activation of NF-kappaB by Toll-like receptor 3. Nature 413, 732–738 10.1038/35099560 11607032

[50] Palou A., Arola L. and Alemany M. (1977) Plasma amino acid concentrations in pregnant rats and in 21-day foetuses. Biochem. J. 166, 49–55 10.1042/bj1660049 901417 PMC1164955

[51] Greizerstein H.B. (1982) Placental and fetal composition during the last trimester of gestation in the rat. Biol. Reprod. 26, 847–853 10.1095/biolreprod26.5.847 7093403

[52] De Miranda J. et al. (2010) Induction of Toll-like receptor 3-mediated immunity during gestation inhibits cortical neurogenesis and causes behavioral disturbances. mBio 1, e00176–e00210 20941330 10.1128/mBio.00176-10 PMC2953007

[53] Koga K. et al. (2009) Activation of TLR3 in the trophoblast is associated with preterm delivery. Am. J. Reprod. Immunol. 61, 196–212 10.1111/j.1600-0897.2008.00682.x 19239422 PMC2765929

[54] Meyer U. et al. (2006) The time of prenatal immune challenge determines the specificity of inflammation-mediated brain and behavioral pathology. J. Neurosci. 26, 4752–4762 10.1523/JNEUROSCI.0099-06.2006 16672647 PMC6674174

[55] Norman N.A. and Bruce N.W. (1979) Fetal and placental weight relationships in the rat at Days 13 and 17 of gestation. J. Reprod. Fertil. 57, 345–348 10.1530/jrf.0.0570345 513025

[56] Vorhees C.V. et al. (2012) Prenatal immune challenge in rats: altered responses to dopaminergic and glutamatergic agents, prepulse inhibition of acoustic startle, and reduced route-based learning as a function of maternal body weight gain after prenatal exposure to poly IC. Synapse 66, 725–737 10.1002/syn.21561 22473973 PMC3370146

[57] Borsini A. et al. (2015) The role of inflammatory cytokines as key modulators of neurogenesis. Trends Neurosci. 38, 145–157 10.1016/j.tins.2014.12.006 25579391

[58] Smith S.E.P., Elliott R.M. and Anderson M.P. (2012) Maternal immune activation increases neonatal mouse cortex thickness and cell density. J. Neuroimmune Pharmacol. 7, 529–532 10.1007/s11481-012-9372-1 22570011 PMC3672058

[59] Wu W.L. et al. (2015) The interaction between maternal immune activation and alpha 7 nicotinic acetylcholine receptor in regulating behaviors in the offspring. Brain Behav. Immun. 46, 192–202 10.1016/j.bbi.2015.02.005 25683697 PMC4414803

[60] Wu W.-L. et al. (2017) The placental interleukin-6 signaling controls fetal brain development and behaviour. Brain Behav. Immun. 62, 11–23 10.1016/j.bbi.2016.11.007 27838335 PMC5373986

[61] Gallagher D. et al. (2013) Transient maternal IL-6 mediates long-lasting changes in neural stem cell pools by deregulating an endogenous self-renewal pathway. Cell Stem Cell 13, 564–576 10.1016/j.stem.2013.10.002 24209760

[62] Hsiao E.Y. and Patterson P.H. (2011) Activation of the maternal immune system induces endocrine changes in the placenta via IL-6. Brain Behav. Immun. 25, 604–615 10.1016/j.bbi.2010.12.017 21195166 PMC3081363

[63] McColl E.R. and Piquette-Miller M. (2019) Poly(I:C) alters placental and fetal brain amino acid transport in a rat model of maternal immune activation. Am. J. Reprod. Immunol. 81, e13115 10.1111/aji.13115 30924965

[64] Ben-Yehuda H. et al. (2020) Maternal type-I interferon signalling adversely affects the microglia and the behaviour of the offspring accompanied by increased sensitivity to stress. Nat. Mol. Psych. 25, 1050–1067 10.1038/s41380-019-0604-0 PMC7192855 31772304

[65] Gilmore J.H., Jarskog L.F. and Vadlamudi S. (2005) Maternal poly I:C exposure during pregnancy regulates TNF alpha, BDNF, and NGF expression in neonatal brain and the maternal-fetal unit of the rat. J. Neuroimmunol. 159, 106–112 10.1016/j.jneuroim.2004.10.008 15652408

[66] Beyaert R. et al. (1996) The p38/RK mitogen-activated protein kinase pathway regulates interleukin-6 synthesis response to tumor necrosis factor. EMBO J. 15, 1914–1923 10.1002/j.1460-2075.1996.tb00542.x 8617238 PMC450110

[67] Smith S.E. et al. (2007) Maternal immune activation alters fetal brain development through interleukin-6. J. Neurosci. 27, 10695–10702 10.1523/JNEUROSCI.2178-07.2007 17913903 PMC2387067

[68] Dahlgren J. et al. (2006) Interleukin-6 in the maternal circulation reaches the rat fetus in mid-gestation. Pediatr. Res. 60, 147–151 10.1203/01.pdr.0000230026.74139.18 16864694

[69] Banks W.A., Kastin A.J. and Gutierrez E.G. (1994) Penetration of interleukin-6 across the murine blood-brain barrier. Neurosci. Lett. 179, 53–56 10.1016/0304-3940(94)90933-4 7845624

[70] Brown A.G. et al. (2017) Exposure to intrauterine inflammation alters metabolomic profiles in the amniotic fluid, fetal and neonatal brain in the mouse. PLoS ONE 12, e0186656 10.1371/journal.pone.0186656 29049352 PMC5648237

[71] Shimomura Y. and Kitaura Y. (2018) Physiological and pathological roles of branched-chain amino acids in the regulation of protein and energy metabolism and neurological functions. Pharmacol. Res. 133, 215–217 10.1016/j.phrs.2018.05.014 29803540

[72] Aye L., Jansson T. and Powell T.L. (2015) TNF-α stimulates System A amino acid transport in primary human trophoblast cells mediated by p38 MAPK signaling. Physiol. Rep. 3, e12594 10.14814/phy2.12594 26508738 PMC4632960

[73] Jones H.N., Jansson T. and Powell T.L. (2009) IL-6 stimulates system A amino acid transporter activity in trophoblast cells through STAT3 and increased expression of SNAT2. Am. J. Physiol. Cell Physiol. 297, C1228–C1235 10.1152/ajpcell.00195.2009 19741197

[74] Mark P.J. et al. (2013) The inflammatory state of the rat placenta increases in late gestation and is further enhanced by glucocorticoids in the labyrinth zone. Placenta 34, 559–566 10.1016/j.placenta.2013.04.006 23639575

[75] Carbó N., López-Soriano F.J. and Argilés J.M. (1995) Administration of tumor necrosis factor-alpha results in a decreased placental transfer of amino acids in the rat. Endocrinology 136, 3579–3584 10.1210/endo.136.8.7628396 7628396

[76] Carbó N. et al. (1996) Tumour growth results in changes in placental amino acid transport in the rat: a tumour necrosis factor alpha-mediated effect. Biochem. J. 313, 77–82 10.1042/bj3130077 8546713 PMC1216912

[77] Bergdolt L. and Dunaevsky A. (2019) Brain changes in a maternal immune activation model of neurodevelopmental brain disorders. Prog. Neurobiol. 175, 1–19 10.1016/j.pneurobio.2018.12.002 30590095 PMC6413503

[78] Ursini G. et al. (2018) Convergence of placenta biology and genetic risk for schizophrenia. Nat. Med. 24, 792–801 10.1038/s41591-018-0021-y 29808008

[79] Ginhoux F. et al. (2013) Origin and differentiation of microglia. Front. Cell. Neurosci. 7, 45 10.3389/fncel.2013.00045 23616747 PMC3627983

[80] Bordeleau M. et al. (2019) Microglia along sex lines: From brain colonization, maturation and function, to implication in neurodevelopmental disorders. Semin. Cell Dev. Biol. 94, 152–163 10.1016/j.semcdb.2019.06.001 31201858

