# Supplementary material for: Maternal immune activation in rats induces dysfunction of placental leucine transport and alters fetal brain growth
Source: Clin Sci (Lond). 2022 Aug 5;136(15):1117–37. doi: 10.1042/CS20220245 (PMC9366863; doi:10.1042/CS20220245)
Supplement: Supplementary Figures S1-S5 and Table S1 [file CS-2022-0245_supp.pdf]

Supplementary Figure 1

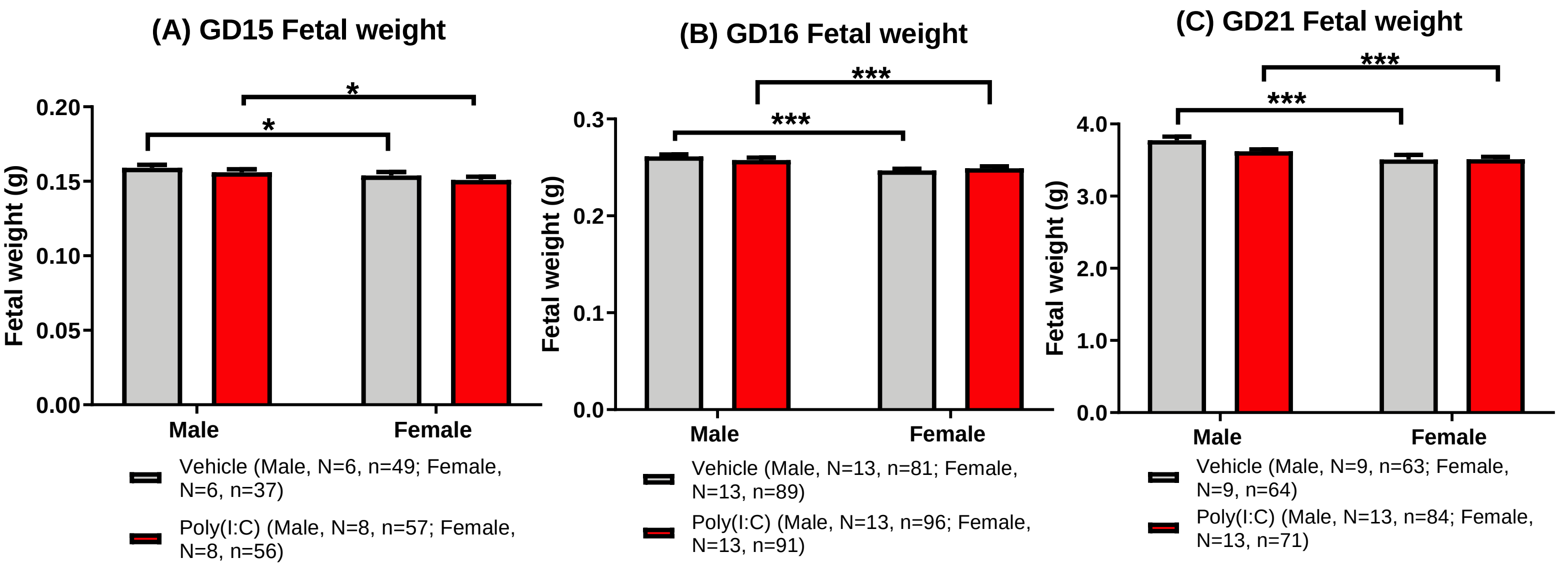

Supplementary Figure 1. Male fetal body weight was heavier than female body weight at all gestational ages and for both treatment groups. (A) GD15, (B) GD16 and **(C) GD21.** Data presented as mean + SEM. *P<0.05, ***P<0.001, with significance displayed for sex-related differences in both treatment groups.

Supplementary Figure 2

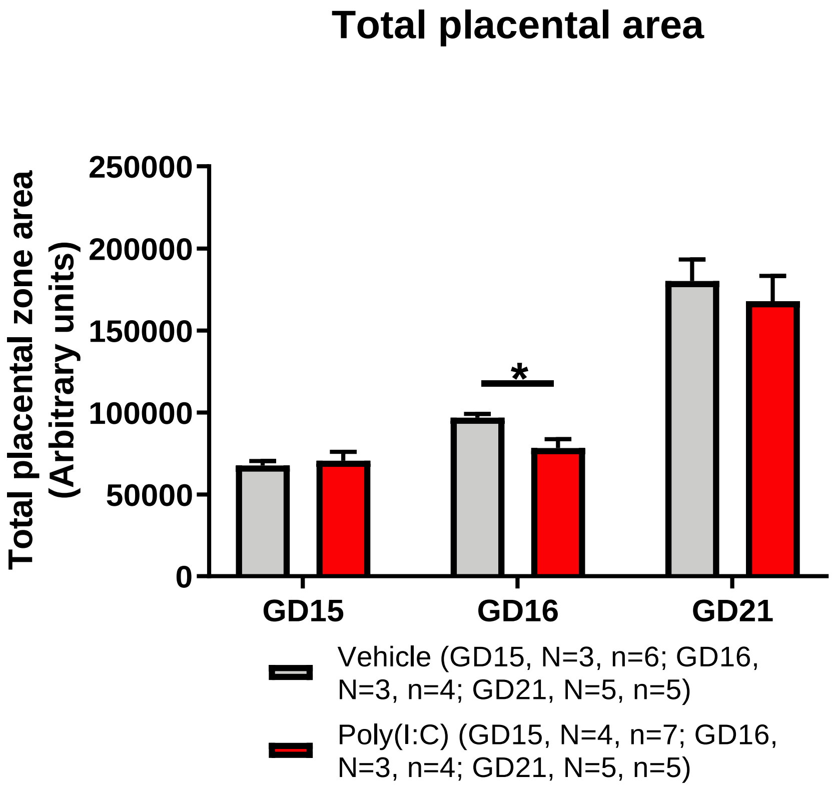

Supplementary Figure 2. Total placental area (combined area of placental junctional and labyrinth zones) at GD15, 16 and 21. Data are presented as mean + SEM, with n referring to the number of placental sections from individual fetuses and N number of litters. *P<0.05.

Supplementary Figure 3

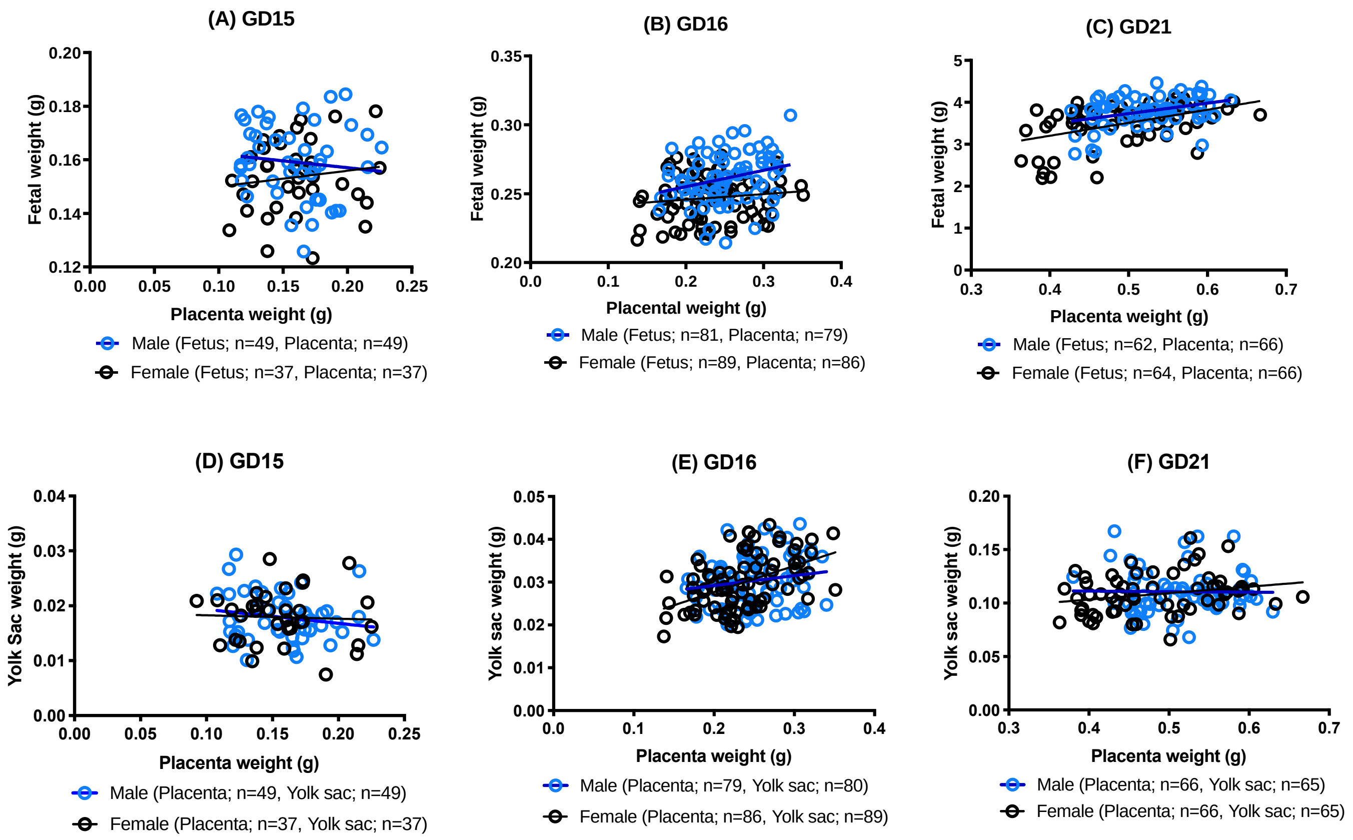

Supplementary Figure 3. (A-C) Relationship between placental weight and fetal weight at (A) GD15, (B) GD16 and (C) GD21. Fetal and placental weights were significantly correlated at GD16 in males (r=0.27, P=0.02), but not females (r=0.12, P=0.26) and at GD21 in both males (r=0.35, P=0.006) and females (r=0.46, P<0.001), but not at GD15 in either males (r=-0.11, P=0.47) or females (r=0.14, P=0.43). **(D-E) Relationship between placental and yolk sac weight at (D) GD15, (E) GD16 and (F) GD21**. At GD15, neither male (r=-0.18, P=0.24) nor female (r=-0.04, P=0.81) fetuses showed a significant correlation between placental and yolk sac weights. Similarly, at GD16 (r = 0.17, P=0.16) and GD21 (r=-0.014, P=0.92), there was no correlation observed between tissue weights for male fetuses. In contrast, there was a significant correlation between placenta and yolk sac weights in female fetuses at GD16 (r=0.48, P<0.0001) and a trend correlation in the female group at GD21 (r=0.24, P=0.063). For GD15, GD16 and GD21, N=6, 13 and 9, respectively. Individual n numbers denote number of male and female fetuses.

Supplementary Figure 4

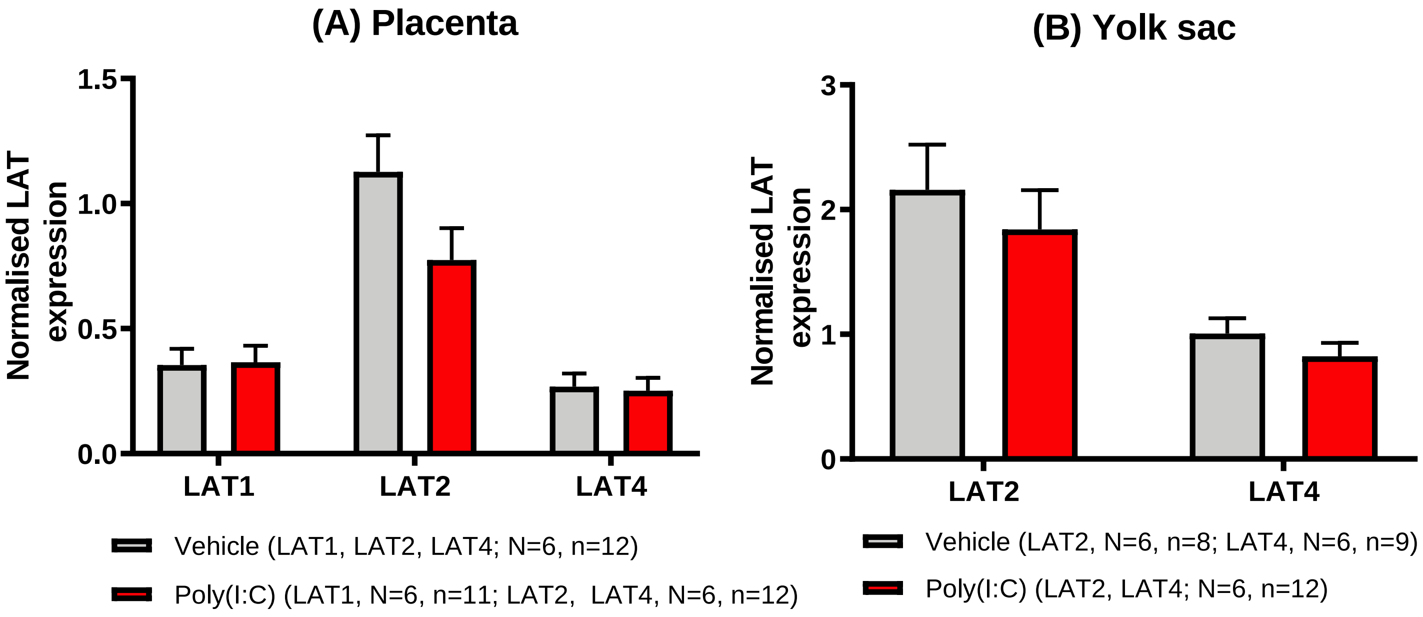

Supplementary Figure 4. LAT protein expression in (A) placenta and (B) yolk sac at GD16. (A) Placental LAT expression. LAT1, LAT2 and LAT4 were each expressed in placenta, with placental LAT1 and placental LAT4 expression unaffected by poly(I:C) treatment. LAT2 exhibited a non-significant trend towards a decrease in the poly(I:C) group (P=0.072). **(B) Yolk sac LAT expression.** Only LAT2 and LAT4 expression was detected in yolk sac at GD16 at comparable protein loadings, so LAT1 is not shown. Yolk sac expression of both LAT2 and LAT4 subtypes was unaffected by treatment. Data presented as mean + SEM.

Supplementary Figure 5

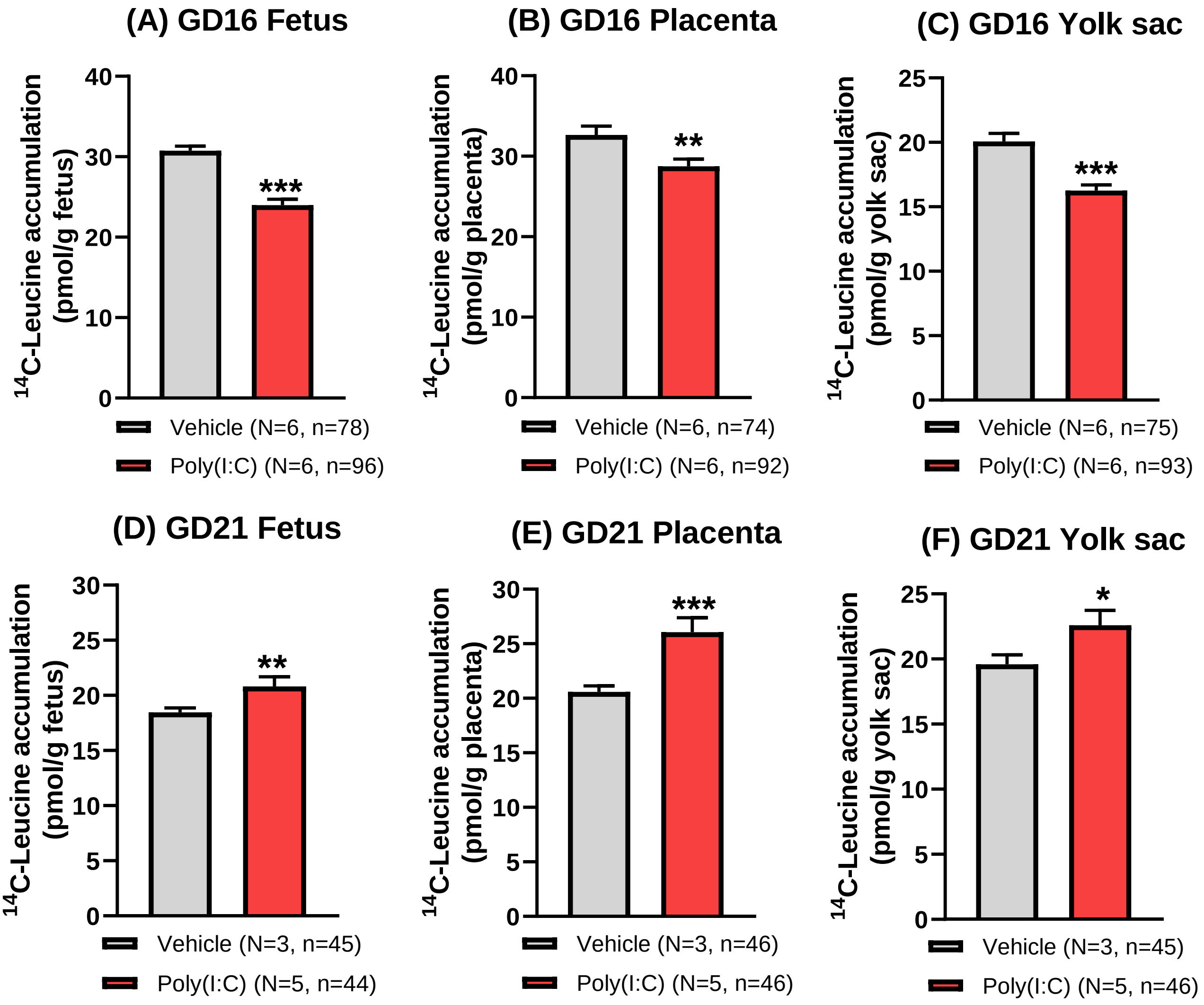

Supplementary Figure 5. Tissue accumulation of ^{14}C-leucine in the fetus, placenta and yolk sac on GD16 (24 h) and GD21 (6 days) post-poly(I:C) treatment. At GD16, **(A)** fetal, **(B)** placental and **(C)** yolk sac accumulation of radiolabelled tracer, ^{14}C-leucine, was significantly reduced in poly(I:C)-treated group compared to vehicle in each tissue compartment. At GD21, **(D)** fetal, **(E)** placental and **(F)** yolk sac accumulation of ^{14}C-leucine, was significantly increased in poly(I:C)-treated group compared to vehicle in each tissue compartment. Data presented as mean + SEM. *P<0.05, **P<0.01, ***P<0.001 vs vehicle control.

Supplementary Table 1. Primers used for gene targets.

**Gene**	**Protein encoded**	**QuantiTect Primer Assay**	**Genbank Accession No.**
Il6	Interleukin-6	QT00182896	NM_012589
Il1b	Interleukin-1β	QT00181657	NM_031512
Tnf	Tumour necrosis factor α	QT00178717	NM_012675
Tlr3	Toll-like receptor 3	QT01603532	NM_198791
Slc7a5	LAT1	QT00188090	NM_017353
Slc7a8	LAT2	QT00193116	NM_053442
Slc43a2	LAT4	QT01604638	NM_001105812
